# COVID-19 and EQ-5D-5L health state valuation

**DOI:** 10.1007/s10198-023-01569-8

**Published:** 2023-02-23

**Authors:** Edward J. D. Webb, Paul Kind, David Meads, Adam Martin

**Affiliations:** 1 Academic Unit of Health Economics, Leeds Institute of Health Sciences, University of Leeds, Leeds, UK; 2 Institute of Epidemiology and Health, University College London, UK and Academic Unit of Health Economics, Leeds Institute of Health Sciences, University of Leeds, Leeds, UK

**Keywords:** COVID-19, EQ-5D-5L, Valuation, Visual analogue scale, Health shock, D7, I10, I30

## Abstract

**Background:**

We investigate whether and how general population health state values were influenced by the initial stages of the COVID-19 pandemic. Changes could have important implications, as general population values are used in health resource allocation.

**Data:**

In Spring 2020, participants in a UK general population survey rated 2 EQ-5D-5L states, 11111 and 55555, as well as dead, using a visual analogue scale (VAS) from 100 = best imaginable health to 0 = worst imaginable health. Participants answered questions about their pandemic experiences, including COVID-19’s effect on their health and quality of life, and their subjective risk/worry about infection.

**Analysis:**

VAS ratings for 55555 were transformed to the full health = 1, dead = 0 scale. Tobit models were used to analyse VAS responses, as well as multinomial propensity score matching (MNPS) to create samples balanced according to participant characteristics.

**Results:**

Of 3021 respondents, 2599 were used for analysis. There were statistically significant, but complex associations between experiences of COVID-19 and VAS ratings. For example, in the MNPS analysis, greater subjective risk of infection implied higher VAS ratings for dead, yet worry about infection implied lower ratings. In the Tobit analysis, people whose health was affected by COVID-19 rated 55555 higher, whether the effect on health was positive or negative.

**Conclusion:**

The results complement previous findings that the onset of the COVID-19 pandemic may have impacted EQ-5D-5L health state valuation, and different aspects of the pandemic had different effects.

## Introduction

The COVID-19 pandemic has had an enormous effect on health and society worldwide. Over six million people have died as of July 2022 [1]. COVID-19 can lead to a variety of sequelae [2, 3], including neurological [4] and cardiac [5] problems, fatigue [6] and mental health effects [7]. The pandemic has had a large economic impact [8] as well as straining healthcare resources [9, 10], diverting care from other areas, worsening outcomes [11, 12], and creating a backlog of patients awaiting treatment [13–15].

It is plausible that disruption from the pandemic has led people to reassess their preferences, attitudes and priorities, including for health and healthcare; for example, what trade-offs they would be willing to make between health and quality of life. The possibility implies changes in how people would value health states and instruments measuring health-related quality of life. In particular, it is important to examine the EQ-5D instrument [16], as in many countries, including the UK [17], national EQ-5D value sets are used to allocate healthcare resources according to the public’s preferences. If people’s values for EQ-5D health states have changed due to the COVID-19 pandemic, the implication is that healthcare resources are not being allocated efficiently, a problem which is particularly acute when such resources are scarcer than ever.

If values for EQ-5D health states have changed due to COVID-19, there are also implications for recent and ongoing valuation exercises. Value sets created just before the pandemic may no longer be valid. Alternatively, if the shock to values is transient, value sets created in the present could have a short shelf life. In general, the “life-cycle” of value sets is a neglected area, and there have been calls for further research into it [18]. Investigating whether the COVID-19 health crisis affected people’s health preferences gives insight into whether it may have brought to an end the useful life of existing national EQ-5D value sets.

This paper examines specifically whether and how the onset of the COVID-19 pandemic affected how individuals from the UK valued EQ-5D-5L, the five-level version of the instrument [19], which measures whether people have problems on five dimensions: mobility, self-care, usual activities, pain/discomfort and anxiety/depression. On each dimension, individuals indicate whether they have no, mild, moderate, severe or extreme problems. Our work is a follow-up to Webb et al. [20], who compared survey data collected in 2018 and in 2020, shortly after the onset of the pandemic in the UK. They examined differences between the two time points in how individuals rated two EQ-5D-5L health states, 11111 and 55555, and dead using a visual analogue scale (VAS), as well as a derived value for 55555 on the full health = 1, dead = 0 scale used to calculate quality adjusted life-years (QALYs). In 2020 compared to 2018, ratings for 11111 and dead were lower, whereas ratings for 55555 were higher, both for the original VAS and the 1–0 scale. There were also differential changes according to subgroups such as gender, ethnicity and age.

Webb et al. [20] propose that the COVID-19 pandemic is the most likely cause of differences between 2018 and 2020. This paper seeks to complement and reinforce the previous findings by examining the survey data collected in 2020 more closely and exploiting its richness in terms of the number of questions asked about people’s COVID-19 experiences. In particular, we use responses to two survey questions about people’s perceived risk/worry about infection, and four questions about how people’s lives were affected during the pandemic, to examine whether people whose lives were more affected change their values more, which would lend credence to the supposition that the pandemic is the driver of value change.

This study uses VAS to measure people’s preferences for heath states, whereas EQ-5D value sets are usually created using time trade-off (TTO), possibly augmented with a discrete choice experiment (DCE) [21]. We comment on the relevance of our result for such value sets in Sect. “Strengths and weaknesses”.

## Methods

### Data collection

**Fig. 1:**
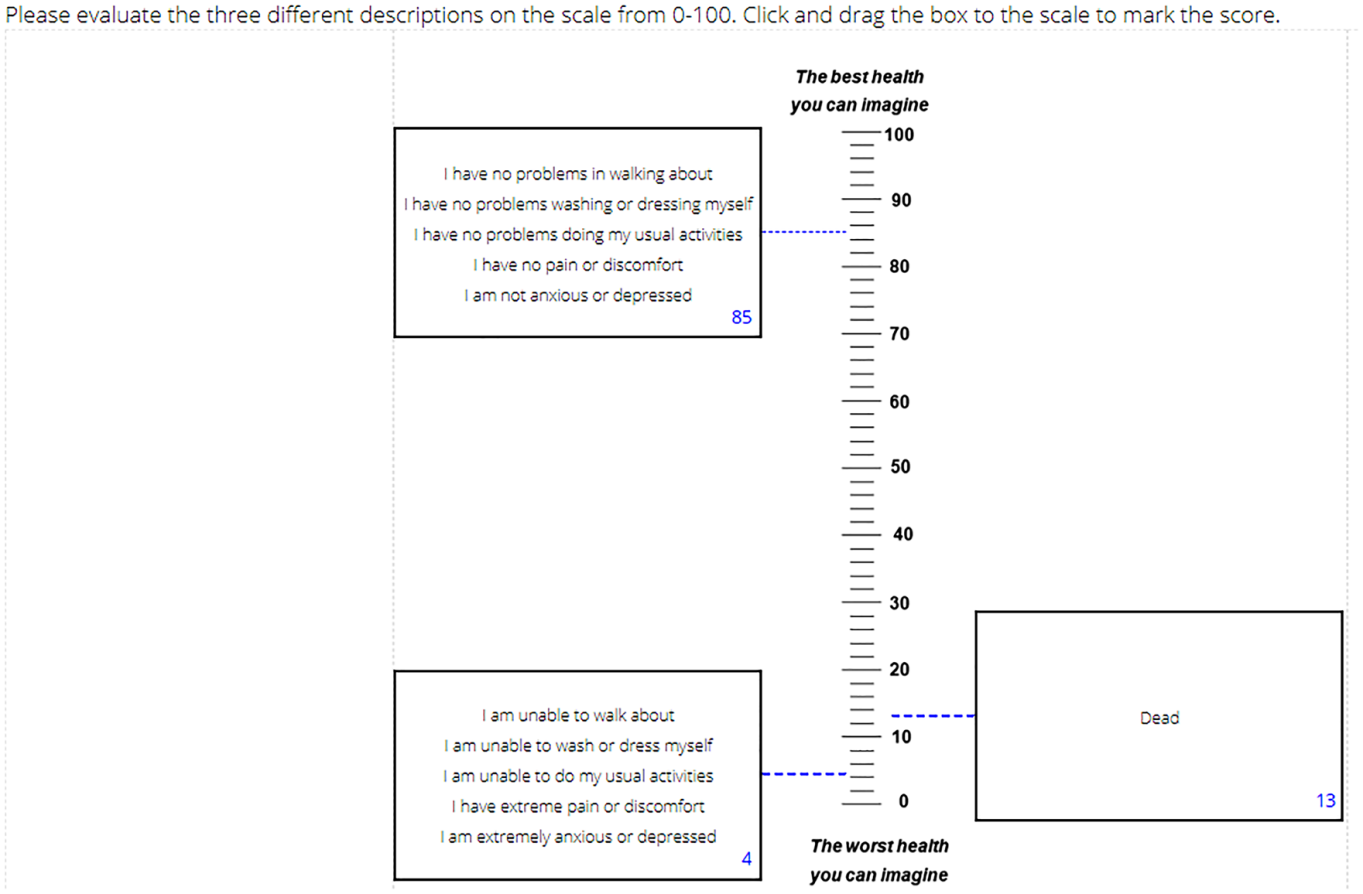
Example of visual analogue scale task

Primary data were collected using an online survey. Recruitment took place in two waves: 16th–23rd April 2020 and 4th–15th May 2020. Some individuals responded to both waves, and some only to one. Participants were asked questions about themselves and their experience of COVID-19. They rated two EQ-5D-5L states, 11111 and 55555, as well as dead, using the VAS task illustrated in Fig. 1. The scale ran from 100 = the best health you can imagine to 0 = the worst health you can imagine.

Participants were asked whether they had received a positive diagnosis or test for COVID-19. Those answering no were asked what they considered their chances of becoming infected with COVID-19 were, as well as whether they worried about being infected with COVID-19, both on Likert scales from 1 to 5. In wave 2, only additional questions were asked whether COVID-19 had affected their health and/or quality of life, with possible answers of no, yes-negatively and yes-positively. Participants were asked how often they left their homes to shop, and to get fresh air and exercise, and whether they considered themselves key workers. A full list of the COVID-19-related questions is given in appendix A. The survey did not allow participants to proceed without answering all questions; therefore, there is no missing data in submitted responses. (Although note as specified above, some questions were not asked in wave 1.)

Secondary data on COVID-19 prevalence and deaths on the final day of each recruitment wave (23/4/20 and 15/5/20) at Nomenclature of Territorial Units for Statistics (NUTS) level 1 were drawn from the UK coronavirus dashboard^1^ and linked to each individual. The specific measures were cumulative COVID-19 cases by date of publication and cumulative deaths within 60 days of a positive test by date of death.^2^

### Analysis

Differences between wave 1 and 2 characteristics were assessed using Mann–Whitney *U* tests.

VAS ratings for 55555 on the 100–0 scale were transformed to the full health = 1, dead = 0 scale used for calculating quality-adjusted life-years (QALYs) [22]. This was done using the formula

$${\text{VAS}}_{{{55555}}}^{{{\text{rescaled}}}} = \frac{{{\text{VAS}}_{{{55555}}} - {\text{VAS}}_{{{\text{dead}}}} }}{{{\text{VAS}}_{{{11111}}} - {\text{VAS}}_{{{\text{dead}}}} }}$$

where ${VAS}_{i}$ is the rating of health state $i$ on the 100–0 scale and ${\text{VAS}}_{{{55555}}}^{{{\text{rescaled}}}}$ is the value of 55555 on the 1–0 scale. The rescaled values of 55555 are dependent on ratings for 11111 and dead. Thus, it is possible for individuals’ rescaled 55555 values to increase in response to COVID-19 while their raw VAS ratings decrease, and vice versa, depending on how their ratings for 11111 and dead also change. The rescaling requires VAS ratings to be logical, i.e. ${\text{VAS}}_{{{11111}}}$ $>$ and ${\text{VAS}}_{{{11111}}} {\text{ > VAS}}_{{{55555}}}$, and illogical responses were excluded from the analysis.

The VAS task can produce a tail of very low rescaled values for 55555, for example below  – 100, which can have a large influence on the mean [22]. In line with the previous study, rescaled values for 55555 were censored at -1.

Few individuals (*N* = 17) reported a formal COVID-19 diagnosis or positive test. Due to the low numbers and the fact that they were not shown key questions about their subjective risk of/degree of worry about COVID-19 infection, they were also excluded from the analysis.

Whether subjective risk of COVID-19 infection and worry about COVID-19 infection differed between the waves was assessed using Mann–Whitney *U* tests.

VAS ratings were analysed using two complementary approaches. Tobit regressions were suitable for analysing the continuous variables of cumulative COVID-19 cases/deaths, as well as allowing for interactions between variables. Multinomial propensity score matching (MNPS) was better able to isolate the causal effects of a given dependent variable, but could not accommodate continuous dependent variables or interaction terms. MNPS also required discarding some information by compressing Likert scale responses from a five- to a three-point scale.

Using pooled wave 1 and wave 2 data, three Tobit regressions were run with VAS ratings of 11111, dead and 55555 as dependent variables and upper limits of 100 and lower limits of 0. A Tobit regression was run with rescaled values of 55555 as the dependent variable and an upper limit of 1 and lower limit of -1 was run. Individuals’ subjective risk of COVID-19 infection, worry about COVID-19 infection and the interaction of the two were included as independent variables, along with cumulative cases and deaths by region and the following controls: age, age^2^, female, white, left school after minimum age, has degree or equivalent qualification, live alone, retired, employed, whether self-report being in levels 2–5 on each EQ-5D-5L dimension, number of long-term health conditions, self-rated health on a Likert scale from 1 to 5.

Using only wave 2 data, similar sets of Tobit regressions were run which added whether COVID-19 had affected participants’ health and/or quality of life. The regressions also included a variable indicating if someone considered themselves a key worker. Two further sets of Tobit regressions were then run which included first how often individuals left the house to shop, and second how often individuals left the house for fresh air and exercise.

There is potential for co-linearity between many variables of interest as well as controls. For example, correlation was expected between subjective risk of infection and worry about COVID-19. Older individuals, or those with long-term health conditions, may also be more concerned about COVID-19, as well as valuing health differently from younger, healthier individuals. MNPS was used to isolate the causal effect of: subjective risk of COVID-19 infection, worry about COVID-19 infection, whether COVID-19 affected health/quality of life, shopping frequency and exercise frequency. The five level responses for subjective risk and worry were collapsed to three groups: 1–2, 3 and 4–5. Responses for frequency of leaving the house for shopping/exercise were collapsed to: less than weekly, weekly and more than weekly. For each variable of interest, samples were created which were balanced on the same control variables used in the Tobit regressions, as well as on the other variables of interest using the twang package for R [23]. Twang estimates multinomial propensity score weights using gradient boosted models, an iterative machine learning technique which can accommodate non-linearity and interactions among the variables that the researcher seeks to achieve balance on. The estimand, i.e. the causal effect of interest, was the average treatment effect (ATE). The gradient boosting algorithm was run for 5,000 iterations, with the multinomial propensity score weights used for analysis taken from the iteration which minimised differences between treatment groups. Differences were assessed using the mean standardised effect size across all control variables. ATEs were then found by including the propensity score weights in weighted linear regression models.

To test the results’ robustness to the rescaled 55555 threshold of  – 1, the Tobit models were re-run with thresholds of  – 2 and  – 1.5. In addition, the models were re-run using a number of other approaches, which rather than censoring, removed participants with excessively low values for 55555 from the analysis data. Full details are given in appendix C, but in summary the approaches were: excluding participants with low rescaled values of 55555; excluding participants who had a large influence on the mean rescaled value of 55555; excluding participants with a high rate of change of rescaled 55555 values with respect to raw 55555 values.

All analysis was carried out using R.

## Results

There were 3021 total responses to the survey, comprising 809 people who responded in wave 1 only, 826 who responded in wave 2 only, and 693 people who participated in both. There were 422 responses (14.0%) excluded for illogical VAS ratings. Only 11 respondents (0.4%) said they had received a positive COVID-19 diagnosis and 6 (0.2%) reported ever having a positive test result, all submitted illogical VAS ratings and were excluded on that basis.

Table 1 summarises the characteristics of both the full (*N* = 3021) and analysis (*N* = 2599) samples and Table 2 summarises their responses to COVID-19-related questions. Both samples were similar, although the analysis sample was slightly older (48.3 years vs. 47.7 years) and were less likely to have a long-term health condition (30.2% vs. 32.7%). There were relatively few older people in the analysis sample, with only 4.1% aged over 75, compared to 8.6% of the UK population [24]. Around 20% of wave 2 participants said that COVID-19 had affected their health negatively, while around 5% said its effect was positive. A larger proportion said COVID-19 had affected their quality of life, with almost half reporting a negative impact and 8.3% reporting a positive impact. The modal frequency of leaving the house to shop was weekly, whereas the modal frequency of leaving the house for exercise and fresh air was daily. Almost no one shopped more than daily (6; 0.5%) and few exercised more than daily (67; 5.1%) (Table 2).

**Table 1:** Participants’ characteristics

	Full sample		Analysis sample			
			Both waves		Wave 2	
	*N*	(%)	*N*	(%)	*N*	(%)
*Age*						
Mean (sd)	47.7	(16.7)	48.3	(16.7)	49.7	(16.1)
18–24	307	(10.2)	243	(9.30)	87	(6.6)
25–34	480	(15.90	398	(15.3)	196	(14.9)
35–44	475	(15.70	413	(15.9)	200	(15.2)
45–54	652	(21.6)	547	(21)	293	(22.2)
55–64	418	(13.8)	367	(14.1)	212	(16.1)
65–74	577	(19.1)	531	(20.4)	276	(20.9)
75 +	112	(3.7)	100	(3.8)	54	(4.1)
*Female*						
	1489	(49.3)	1285	(49.4)	648	(49.2)
*Ethnicity*						
White	2694	(89.2)	2337	(89.9)	1190	(90.3)
Asian	130	(4.3)	106	(4.10	56	(4.2)
Mixed	51	(1.7)	39	(1.5)	19	(1.4)
Black	88	(2.9)	74	(2.8)	33	(2.5)
Other	58	(1.9)	43	(1.7)	20	(1.5)
*Occupation*						
Employed	1789	(59.2)	1554	(59.8)	799	(60.6)
Key worker*	519	(34.2)	–	**–**	477	(36.2)
Retired	653	(21.6)	592	(22.8)	307	(23.3)
Housework	204	(6.80)	167	(6.4)	82	(6.2)
Student	108	(3.6)	93	(3.60	34	(2.6)
Unemployed	123	(4.1)	95	(3.70)	49	(3.7)
Prefer not to say	58	(1.9)	40	(1.50)	15	(1.1)
Other	86	(2.80	58	(2.20)	32	(2.4)
*Education*						
Left school after minimum age	2330	(77.1)	2025	(77.9)	1026	(77.8)
Degree/ equivalent	1582	(52.4)	1368	(52.6)	703	(53.3)
Report being in 11111	1037	(34.3)	936	(36)	481	(36.5)
EQ-VAS	73	(22.5)	74.7	(21.1)	74.8	(21.1)
Long-term condition	987	(32.7)	786	(30.2)	402	(30.5)
*Number of comorbidities*						
Mean (sd)	0.7	(1.20)	0.6	(1.20)	0.6	(1.1)
*Description of own health*						
Excellent	357	(11.8)	306	(11.8)	145	(11)
Very good	1028	(340)	929	(35.7)	478	(36.3)
Good	990	(32.8)	872	(33.6)	436	(33.1)
Fair	507	(16.8)	398	(15.3)	210	(15.9)
Poor	139	(4.6)	94	(3.6)	49	(3.7)
*N*	3021		2599		1318	

Note. *Only asked in wave 2, *sd * standard deviation

**Table 2:** Responses to COVID-19-related questions

	Full sample		Analysis sample			
			Both waves		Wave 2	
	*N*	(%)	*N*	(%)	*N*	(%)
*COVID-19 diagnosis*	11	(0.4)	0	–	0	–
COVID-19 positive test	6	(0.2)	0	–	0	–
*Worry about catching COVID-19 (1 = never thought about it, 5 = Worried all the time)*						
Mean (sd)	2.2	(1.0)	2.2	(1.0)	2.1	(1.0)
*Subjective risk of COVID-19 infection (1 = highly unlikely, 5 = highly likely*						
Mean (sd)	2	(0.9)	2	(0.9)	2	(0.9)
COVID-19 affected health?*						
Yes, negatively	328	(24.9)	–	–	260	(19.7)
No	1106	(72.8)	–	–	991	(75.2)
Yes, positively	85	(6.4)	-	-	67	(5.1)
COVID-19 affected quality of life?*						
Yes, negatively	729	(48.0)	–	–	641	(48.6)
No	650	(49.3)	–	–	567	(43.0)
Yes, positively	140	(9.2)	–	–	110	(8.3)
Frequency leave house to shop*						
Never	236	(17.9)	–	–	198	(15.0)
Less than weekly	275	(18.1)	–	–	245	(18.6)
Weekly	650	(49.3)	–	–	573	(43.5)
2–6 times per week	285	(18.8)	–	–	246	(18.7)
Daily	62	(4.7)	–	–	50	(3.8)
More than daily	11	(0.7)	–	–	6	(0.5)
Frequency leave house for exercise*						
Never	175	(13.3)	–	–	146	(11.1)
Less than weekly	165	(10.9)	–	–	138	(10.5)
Weekly	191	(14.5)	–	–	152	(11.5)
2–6 times per week	440	(29.0)	–	–	393	(29.8)
Daily	469	(35.6)	–	–	422	(32.0)
More than daily	79	(5.2)	–	–	67	(5.1)
*N*	3021		2599		1318	

Note. *Only asked in wave 2, *sd* standard deviation

Table 5 shows the results of comparing the demographics of waves 1 and 2. The only significant difference observed was in age, with wave 2 participants being slightly older than wave 1 at 45.7 compared to 47. There were no significant differences between survey waves in subjective risk of COVID-19 infection (Mann–Whitney *U* *p* value 0.189) or worry about COVID-19 infection (Mann–Whitney *U* *p* value 0.097).

**Fig. 2:**
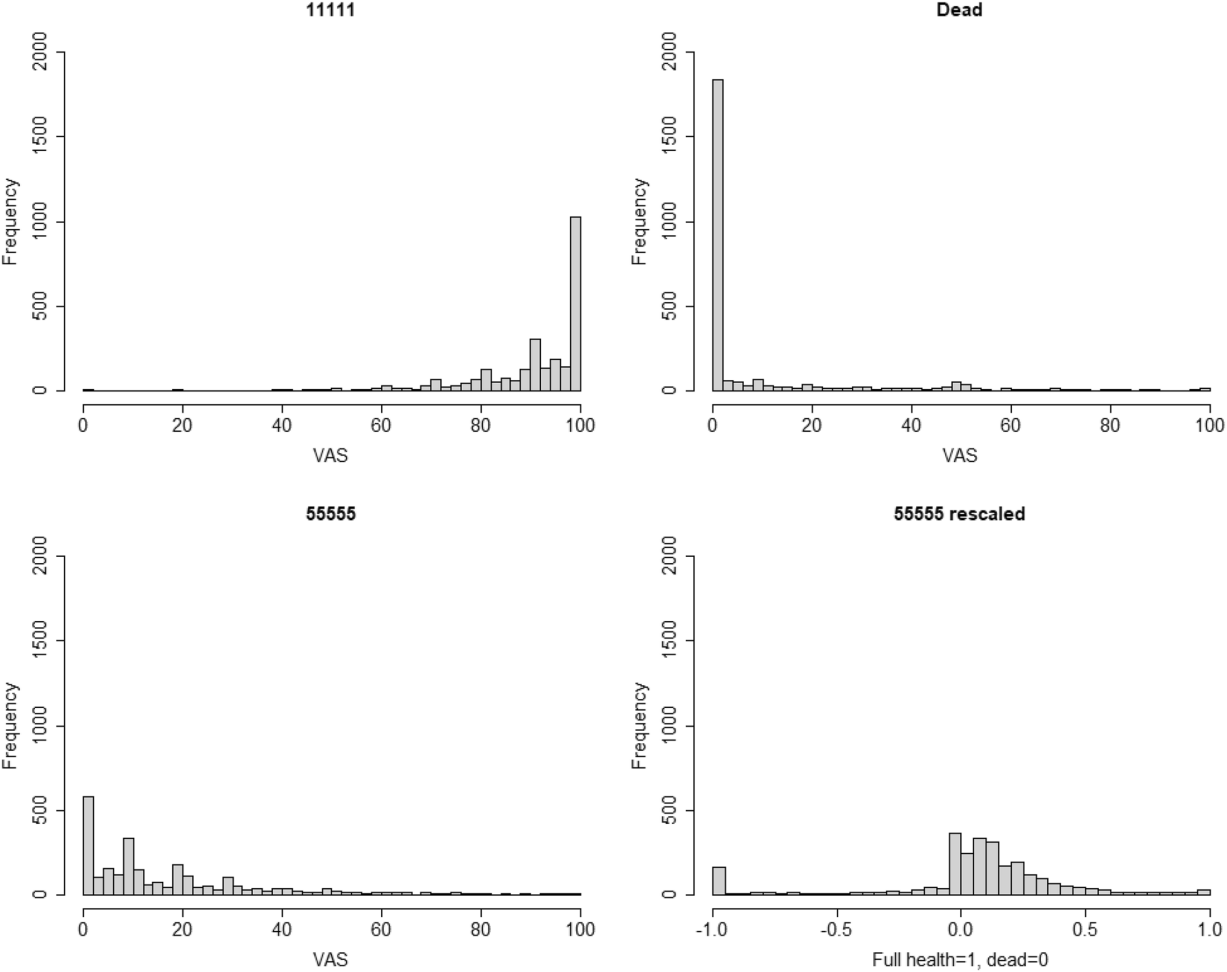
Histograms of VAS responses

Figure 2 shows histograms of the analysis sample’s VAS responses. A large proportion of respondents rated full health as 100 and dead as 0. Most rescaled 55555 values were positive but low, although many also rated it below 0 (i.e. worse than dead).

Table 3 summarises the Tobit model results, with full results including control variables given in Appendix B. With the whole analysis sample, there was no significant main effect of worry about COVID-19 infection on any VAS responses, but there was a significantly positive main effect of subjective risk of COVID-19 infection and a significantly negative interaction with worry for dead and rescaled 55555. There were no significant effects of regional-level COVID-19 cases or deaths. With wave 2 respondents, there were no significant results for COVID-19’s effect on quality of life, but respondents reporting both negative and positive impact of COVID-19 on health rated 55555 significantly higher. There was a similar result for dead, although only the coefficient for positive impact of COVID-19 on health was significant. Frequency of leaving the house for shopping had no significant coefficients. Those who left the house to exercise less than weekly or weekly rated 11111 significantly lower and dead significantly higher compared to never leaving the house for exercise. Those who exercised weekly also rated 55555 significantly higher. Examining all exercise-related coefficients seems to indicate that the effects of exercise frequency on VAS ratings were not linear. For all sets of Tobit regressions using wave 2 data, the effects of subjective risk and worry about COVID-19 infection were similar to results using both waves, although some coefficients no longer achieved statistical significance. COVID-19 cases and deaths had no significant effects.

**Table 3:** Results from Tobit regressions of visual analogue scale ratings

Regression set	Dependent variable	11111	s.e	Dead	s.e	55555	s.e	55555 rescaled	s.e
Both waves * N* = 2599	Cumulative COVID-19 cases	– 0.013	0.0161	0.0399	0.0417	5.85 × 10–3	0.0203	– 1.25 × 10–4	3.72 × 10–4
	Cumulative COVID-19 deaths	0.118	0.0712	– 0.248	0.184	– 0.0431	0.0897	9.33 × 10–4	1.65 × 10–3
	Worry about being infected with COVID-19								
	(1 = never thought about it, 5 = all the time)	0.0331	0.823	0.101	2.12	– 0.827	1.03	– 0.0158	0.0189
	Subjective risk of COVID-19 infection								
	(1 = highly unlikely, 5 = highly likely)	– 0.847	0.862	7.71*	2.16	1.98	1.07	– 0.0578*	0.0198
	Worry × subjective risk	0.206	0.351	– 2.12*	0.9	– 0.0427	0.438	0.0193*	8.08 × 10–3
Wave 2—COVID-19 affected health/quality of life * N* = 1294									
	Cumulative COVID-19 cases	– 8.01 × 10–3	0.0198	0.0318	0.0498	– 4.29 × 10–4	0.0251	– 3.43 × 10–4	– 3.77 × 10–4
	Cumulative COVID-19 deaths	0.12	0.0946	– 0.0901	0.239	– 0.0342	0.12	5.88 × 10–4	7 × 10–4
	Worry about being infected with COVID-19								
	(1 = never thought about it, 5 = all the time)	1.37	1.12	– 2.19	2.81	– 1.57	1.42	– 0.0198	0.0251
	Subjective risk of COVID-19 infection								
	(1 = highly unlikely, 5 = highly likely)	0.0996	1.15	3.97	2.77	0.537	1.45	– 0.0543*	0.0258
	Worry × subjective risk	– 0.223	0.473	– 1.06	1.17	0.0805	0.596	0.0167	0.0106
	COVID-19 affected health (baseline no)								
	Negatively	– 0.154	1.5	4.67	3.71	4.74*	1.89	0.0168	0.0337
	Positively	1.33	2.55	14.8*	5.84	6.18*	–	–	0.0566
	COVID-19 affected quality of life (baseline no)								
	Negatively	0.304	1.15	– 2.96	2.94	– 0.993	1.46	– 9.71 × 10–3	0.0258
	Positively	– 3.54	2.09	8.5	5	0.804	2.64	– 0.0763	0.0471
Wave 2—shopping frequency * N* = 1294									
	How often go shopping (baseline never)								
	< weekly	– 1.56	1.67	1	4.22	– 1.22	2.15	– 0.0111	0.0368
	Weekly	– 0.882	1.47	– 2.92	3.76	0.0746	1.89	3.53 × 10–3	0.0324
	2–6 times/week	– 1.9	1.67	6.9	4.2	– 0.227	2.16	– 0.0415	0.0369
	Daily	– 5.04	2.68	10.4	6.58	5.03	3.49	0.0116	0.0604
	> daily	– 0.844	7.05	5.83	17	15.7	8.89	0.201	0.157
Wave 2—exercise frequency * N* = 1294									
	How often exercise (baseline never)								
	< weekly	– 4.29*	2.02	12.2*	5.04	3.89	2.61	– 0.0155	0.0449
	Weekly	– 4.65*	1.98	14.1*	4.96	7.22*	2.56	– 0.0251	0.0442
	2–6 times/week	– 1.68	1.69	1.68	4.37	2.59	2.18	0.0255	0.0374
	Daily	– 2.15	1.69	5.28	4.36	2.78	2.19	– 5.47 × 10–3	0.0373
	> daily	2.59	2.59	6.67	6.5	0.531	3.31	– 0.0131	0.0563

Note. *s.e.* standard error, * significant at 5% level; full results including controls in Appendix B

Table 4 shows how balanced responses were across various variables of interest, both with and without propensity score weights. It can be seen in all cases that MNPS improved balance, although the results for COVID-19’s effect on health is notably worse compared to other variables of interest (1.09, compared to the next worst being 0.268). Table 4 also presents the results of Tobit regression models following MNPS. For dead, the coefficients for subjective risk of and worry about COVID-19 infection had opposite signs, in line with the negative interaction term seen in the Tobit regression models. Subjective risk had a significantly positive effect on dead and 55555 ratings, whereas worry had a significantly negative impact on dead and a positive effect on rescaled 55555. People who reported COVID-19 had affected their health, either positively or negatively, rated dead significantly higher. A similar result was seen for 55555, although only the coefficient for a negative effect was significant. The only significant coefficient for COVID-19’s effect on quality of life was that those reporting a positive impact rated 11111 lower. There were no significant effects for either shopping or exercise frequency.

**Table 4:** Average treatment effects of COVID-19-related variables on visual analogue scale ratings using matched data

	Mean standardised effect size		Effective sample size	11111	s.e	Dead	s.e	55555	s.e	55555 rescaled	s.e
	Pre-matching	Post-matching									
*Subjective risk of COVID-19 infection*											
1–2	0.87	0.161	2200	–	–	–	–	–	–	–	*** – ***
3				– 0.681	0.748	1.75	0.984	1.99 *	1	– 8.6 × 10–3	0.0194
4–5				– 1.43	0.909	4.53 *	1.4	4.47 *	1.32	-5.8 × 10–3	0.0253
*Worry about being infected with COVID-19*											
1–2	0.817	0.168	2094	–	–	–	–	–	–	–	–
3				1.38	0.784	– 4.5 *	1.39	0.126	1.08	0.0727 *	0.0243
4–5				0.894	0.878	– 5.6 *	1.44	– 0.723	1.23	0.0766 *	0.0266
*COVID-19 affected health*											
Yes-negatively	1.964	1.088	1043	– 1.4	1.16	2.97 *	1.48	6.61 *	1.59	0.0316	0.0329
No				–	–	–	–	–	–	–	–
Yes-positively				0.94	1.54	11 *	4.55	2.59	3.82	– 0.141	0.103
*COVID-19 affected quality of life*											
Yes-negatively	1.575	0.268	1050	4.37 × 10–3	0.769	– 0.345	1.08	– 0.407	1.11	– 8.42 × 10–3	0.0226
No				–	–	–	–	–	–	–	–
Yes-positively				– 4.09 *	1.91	3.23	2.09	2.36	2.07	0.0122	0.0396
*How often go shopping*											
< Weekly	0.812	0.23	1132	–	–	–	–	–	–	–	–
Weekly				0.921	0.862	– 1.16	1.31	0.54	1.2	0.0119	0.0254
> Weekly				– 1.37	1.17	2.48	1.69	2.35	1.6	1.64 × 10–4	0.0335
*How often exercise*											
< Weekly	0.672	0.184	1096	–	–	–	–	–	–	–	–
Weekly				– 0.876	1.36	3.35	2.35	2.24	2.36	– 0.0142	0.0464
> Weekly				0.439	0.913	– 1.86	1.65	– 1.13	1.85	0.0161	0.0321

Note. *s.e.*  standard error, *significant at 5% level

The results of robustness tests of different ways of handling extremely low rescaled 55555 values are given in Appendix C. In most cases, alternative approaches reproduce similar results to those presented in the main body of the paper. However, the significant influence of worry and subjective risk on rescaled 55555 values is not reproduced in around half the robustness analyses. Similarly, the finding that those reporting a negative influence of COVID-19 on quality of life rated 55555 lower was not found in several alternative analyses.

## Discussion

There are indications in our results that how individuals value health was influenced by the onset of the COVID-19 pandemic. Yet it is also clear that the reasons/drivers for this finding are complex, and people’s reactions to different aspects of the pandemic are difficult to untangle. For example, several COVID-19-related variables had a significant effect on some health states, but not others, and it is difficult to understand why. There is also evidence that subjective risk of and worry about COVID-19 infection had opposite effects for both dead and rescaled 55555. The differing impact of people’s self-assessed likelihood of catching COVID-19 infection and their worry about infection may be due to the danger the disease poses depending heavily on factors such as age and co-morbidities [25–27]. Supporting this hypothesis, worry about COVID-19 infection was significantly correlated with age (correlation 0.08, Pearson’s test *p* value < 0.001) but subjective risk of infection was not (correlation 0.02, Pearson’s test *p* value 0.225).

In several instances, those reporting that COVID-19 had affected their health gave significantly different responses compared to those who reported no effect. However, the direction of change was the same whether the effect of COVID-19 was positive or negative. One interpretation is that individuals’ health being affected either way by a pandemic disease which dominated public life caused them to reassess their views on health. Another possibility is that the result is due to a small sample size, as only around 5% of respondents said COVID-19 has positively affected their health. This group also likely differed systematically from the general population, since positive health impacts were likely due to factors such as avoiding an arduous commute and/or an unpleasant workplace. People who said COVID-19 positively affected their health were 81.3% employed/self-employed, compared to 60.2% for other individuals, and were also younger, with an average age of 38.8 compared to 49.8 for other respondents, lending weight to the supposition. While MNPS can theoretically eliminate differences in observed characteristics between groups, the worst balancing performance was seen for COVID-19’s impact on health, largely due to the small number reporting a positive effect.

While there were no significant correlations between VAS ratings and how often people left the house for shopping, there were significant and non-linear effects for exercise in the Tobit regressions in Table 3. These effects are difficult to interpret, due to it being unclear how large an impact the pandemic had on respondents’ behaviour. COVID-19 may have influenced how often people leave the houses for fresh air and exercise in different ways: Many will have gone out less to mitigate risk of infection, yet others may have exercised outside more, e.g. due to previously preferring alternative leisure activities. It should also be noted that although the signs of the exercise coefficients in MNPS also reflected a non-linear effect, they were not significant.

No significant effects were seen for regional COVID-19 cases or deaths. This could be due to people’s experience of the pandemic being driven either by national level reporting, or by cases reported in much smaller geographic areas, with regional variations of less importance.

Examining the magnitudes of the significant effects, there were some large effects for VAS ratings. For example, in the Tobit analysis, those whose health was positively affected by COVID-19 rated dead 14.8 points higher than those who did not, a difference covering around a seventh of the VAS range from 0 to 100. However, differences in rescaled values of 55555 were typically small, with magnitudes between 0.005 and 0.07. This is comparable to the smallest utility decrement in Devlin et al.’s [28] English EQ-5D-5L value set at 0.05. Thus, it may be that any changes to how people value health due to COVID-19 on the full health = 1, dead = 0 scale are relatively small.

Comparing the Tobit and MNPS results, in many cases, they are in agreement in terms of the significance and sign of coefficients. Where this is not the case, it is almost always a significant result using one approach, and an insignificant coefficient with the same sign using the other. Thus, it does not appear that using one approach leads to radically different conclusions than using the other.

It was difficult to determine a causal effect of COVID-19 on health state valuation. It may be that individuals who were more affected by the pandemic also systematically valued health differently from people who were less affected. Including control variables in the Tobit regressions and MNPS analysis mitigates this possibility to some extent. However, they can only control for the influence of observable characteristics. It is plausible that unmeasured personality traits, for example, with risk tolerance, could be associated both with health state valuation and measures of COVID-19’s impact such as subjective risk/worry about infection.

The results presented here represent a stronger (though by no means conclusive) argument for a causal influence of COVID-19 on health-state valuation when viewed as complementary to previous findings in Webb et al. [20]. That work showed significant differences in VAS ratings between before (2018) and during the pandemic (2020), and proposed that the pandemic is the most likely cause. And, that some COVID-19-related variables are significantly correlated in this study with VAS responses gives weight to this proposal.

The findings of the two studies are not always in the same direction. For example, in Webb et al. [20], VAS ratings for dead were lower in 2020 than in 2018, yet here, subjective risk of infection and COVID-19 affecting health lead to higher ratings. This is not necessarily contradictory, however. Some COVID-19-related variables, such as worry about infection, were shown in this study to have a negative effect on VAS ratings for dead, and in the previous study, some subgroups rated dead higher in 2020 than in 2018. As there are complicated interactions between individual characteristics and COVID-19 can affect people indifferent ways, it is not easy to compare the two studies’ results.

This study only presents evidence from the initial phase of the pandemic in the UK. Whether any acute changes to health state valuations persist is an unresolved question. The question will be addressed in part by the ongoing national UK EQ-5D-5L valuation study, in preparation since before the pandemic.^3^ However, in most other countries, national valuation studies are not planned in the near future.

Our analysis sample was not representative of the UK population. In particular, it is difficult to tell how representative our respondents were in terms of the dependent variables of interest. For example, while there are other survey studies measuring risk and/or worry attitudes in the UK at a similar time (e.g. [29–31]), none used exactly the same measures. Different question phrasing and scales make it difficult to compare our participants’ attitudes to those reported elsewhere. Thus, it would not be possible to “re-adjust” existing UK value sets using our results. Another reason why such re-adjustment would not be possible is that the analysis sample is not representative in demographic terms, and the Tobit analysis revealed that demographic factors influenced VAS ratings. For example, female respondents rated 11111 higher and 55555 lower than male respondents and white respondents rated dead lower than non-white respondents.

That demographic characteristics affected VAS ratings raises the question as to whether COVID-19 may have had different impacts among different demographic groups. Investigating this possibility would be a useful topic for future research.

### Policy implications

It is unclear whether knowledge about acute changes to health state values would have had a meaningful impact on how public money was spent in the first few months of the pandemic [32]. While some retrospective analyses of COVID-19 spending have been conducted [33–36], it is not clear that measures of cost-effectiveness were a factor in decision-making in the initial crisis stages [37–40]. Ultimately whether decision-making in an acute crisis should take account of cost-effectiveness measures such as maximising QALY gain per pound spent is a question for policymakers (and by extension the voters who appoint them in a democratic society). Yet if anyone should wish to advocate for a larger role of health economics or health state values in future crises, it is essential to have a firm understanding of, and evidence base for, the validity of health economic techniques in such crises.

We present evidence relating to the COVID-19 pandemic only, and not from any other crisis event which could influence values. Yet that one crisis can influence preferences for health states should be a prompt to investigate to what extent other crises could also affect how people value health.

In the longer term, large-scale EQ-5D valuation studies are resource intensive, so it is impractical to construct new ones in every country in the wake of COVID-19, or after every political, historical or health crisis event that may or may not affect how people think about and value health. Many allocation decisions are long term, such as approving health technologies for use for the foreseeable future. It is not clear that such decisions should be influenced by any short-term preference fluctuations.

Nevertheless, our findings suggest the need to develop standardised, resource-light methods to assess whether and how national EQ-5D value sets still reflect people’s preferences, attitudes and values. Such methods could involve collecting smaller amounts of data than full-scale national value sets, or using less resource intensive methods such as online self-complete surveys. Another approach could be re-analysing existing data (see Webb and Kind [41] for a tentative step in this direction).

### Strengths and weaknesses

This study has several strengths. As far as we are aware, this paper and our previous one are the only studies to address whether a large-scale crisis event has affected population health state valuations. Data collection was timely, giving insight into how people valued health shortly after the onset of the COVID-19 pandemic in the UK. We also used complementary analysis techniques: MNPS increased the possibility of identifying causal effects for individual variables, enabling us to disentangle the various effects of different aspects of the pandemic, while the Tobit regressions allowed for continuous dependent variables and interaction terms, as well as exploiting the full variation in the data without compressing responses into coarser categories.

The VAS task asked individuals to give an explicit value for dead, relative to the worst health they can imagine, in contrast to methods such as time trade-off where dead is always assigned a value of 0. This allows us to investigate individuals’ attitudes to health and death, and to what extent people believe any health states are worse than dead. The survey collected several different COVID-19-related variables. This has allowed us to distinguish between different aspects of how the pandemic has affected people.

This study also has several weaknesses. For example, some results were not robust to using different methods to censor or remove extremely low rescaled 55555 values. We valued health states using VAS, whereas national value sets are more usually created using other techniques such as TTO [21]. Thus, it may be that our findings would not have been robust to using a more standard technique. Yet if VAS and TTO both elicit the same underlying health preferences, changes found using one technique should be expected to be found using the other.

The only EQ-5D-5L state for which values on the full health = 1, dead = 0 scale was found was 55555. While this state has theoretical importance as the worst state in the classification system, few people report being in it. Thus, it is difficult to say what impact COVID-19 would have on more commonly experienced health states. Age is a large risk factor in COVID-19 mortality and severe illness [42–44], so it is older people where the largest effects might be expected to be seen. However, the survey collected relatively few older respondents, with, for example, only 4.1% of the analysis sample aged 75 or older.

## Conclusion

We presented evidence that the COVID-19 pandemic may have affected how people value health, as was also seen in Webb et al. [20]. However, there were differences in what aspects of the pandemic influenced individuals’ values. For example, there were differences between people who did and did not report that COVID-19 had affected their health, but no analogous finding for its effect on quality of life. Future research is required to disentangle the complex situation.

Future research could also investigate the influence on valuation of other important health events, or political/social shocks. It would also be useful to investigate whether any effects on valuation, due to COVID-19 or other events, are permanent or transient.

Finally, our results suggest the need to develop standardised methods to quickly and easily assess whether national EQ-5D value sets still represent national values.

## Data Availability

Data may be made available upon request to the corresponding author or Leeds Institute of Health Sciences provided appropriate ethical approvals and data sharing agreements are put in place.

## Appendix

### Appendix A COVID-19-related survey questions

Do you worry about being infected with the Corona virus?

**Table:**

1	2	2	4	5
Never thought about it	Thought about it, but not worried	Worried me a bit	Worried me a lot	Worried about it all the time

What do you think are your chances of becoming infected with the Corona virus?

**Table:**

1	2	3	4	5
Highly likely	Likely	Equally likely or unlikely	Unlikely	Highly unlikely

Note: This variable was re-coded for analysis so higher numbers mean higher subjective risk

Over the past couple of weeks has the COVID-19 pandemic affected your health?

- Yes—it has affected my health for the better
- Yes—it has affected my health for the worse
- No—it has not affected my health

Over the past couple of weeks has the COVID-19 pandemic affected your quality of life?

- Yes—my quality of life has changed for the better
- Yes—my quality of life has changed for the worse
- No—it has not affected my quality of life

Since the start of the COVID-19 lockdown, how often do you leave home to buy food or other essentials?

- Not at all / never
- Less than once a week
- Once a week
- 2–6 times a week
- Once a day
- Several times a day

Since the start of the COVID-19 pandemic, how often do you go outside for fresh air and exercise?

- Not at all / never
- Less than once a week
- Once a week
- 2–6 times a week
- Once a day
- Several times a day

### Appendix B Comparison of wave 1 and wave 2 demographics

See below Appendix, Table 5 here.

**Table 5:** Comparison of wave 1 and wave 2 demographics

	Wave 1 *N*	Wave 2 *N*	*p* value
*Age*			
Mean (standard deviation)	47	49.7	< 0.001
*Female*			
	637	648	0.775
*Ethnicity*			
White	1147	1190	0.397
Asian	50	56	
Mixed	20	19	
Black	41	33	
Other	23	20	
*Occupation*			
Employed	755	799	0.22
Retired	285	307	
Housework	85	82	
Student	59	34	
Unemployed	46	49	
Missing	25	15	
Other	26	32	
*Education*			
Left school after minimum age	999	1026	0.931
Degree or equivalent qualification	665	703	0.467
In 11111	455	481	0.605
EQ-VAS	74.5	74.8	0.732
Long-term condition	384	402	0.771
*Number of comorbidities*			
Mean (standard deviation)	0.644	0.605	0.963
*Description of own health*			
Excellent	161	145	0.393
Very good	451	478	
Good	436	436	
Fair	188	210	
Poor	45	49	
*N*	1281	1318	

Note. *p*-values for Mann–Whitney *U* tests

### Appendix C Full Tobit results

See below Appendix, Tables 6, 7, 8 and 9 here.

**Table 6:** Tobit regression results for both waves

	11111	s.e	Dead	s.e	55555	s.e	55555 rescaled	s.e
Constant	99.2*	4.03	16.7	10.2	19.1*	5.04	– 0.0935	0.0927
Age	– 0.0662	0.152	– 0.501	0.389	0.167	0.191	5.82 × 10–3	3.5 × 10–3
Age^2^	3.68 × 10–4	1.65 × 10–3	2.46 × 10–3	4.3 × 10–3	– 3.68 × 10–3	2.1 × 10–3	– 5.56 × 10–5	3.83 × 10–5
Female	3.27*	0.774	– 3.67	2	– 3.68*	0.975	– 0.011	0.0179
White	4.03*	1.27	– 17.9*	3.08	– 5.62*	1.58	0.0787*	0.0294
*Education*								
Left school after minimum age	1.57	1.1	– 6.97*	2.9	– 0.864	1.4	8.59 × 10–3	0.0256
Degree	– 0.946	0.946	7.11*	2.49	0.694	1.19	– 0.0192	0.0218
Live alone	0.774	0.984	– 4.35	2.59	– 2.96*	1.25	– 9.81 × 10–6	0.0228
*Occupation*								
Retired	– 0.864	1.62	3.5	4.25	1.61	2.06	– 0.0347	0.0376
Employed	– 1.66	1.11	1.8	2.83	0.381	1.39	– 1.49 × 10–3	0.0257
*EQ-5D*								
Mobility levels 2–5	– 0.217	1.35	– 3.11	3.51	2.49	1.71	0.0695*	0.0316
Self-care levels 2–5	– 0.759	1.65	7.11	4.16	3.7	2.09	– 0.0291	0.0388
Usual activities levels 2–5	– 2.22	1.38	6.9	3.53	3.27	1.75	– 0.0136	0.0323
Pain/discomfort levels 2–5	0.188	0.941	3.78	2.43	0.837	1.19	– 0.0249	0.0218
Anxiety/depression levels 2–5	– 2.01*	0.862	5.11*	2.23	1.74	1.09	– 0.0153	2 × 10–2
Number of co-morbidities	0.707	0.399	0.465	1.02	– 0.433	0.505	– 0.0114	9.3 × 10–3
Description of own health	– 3.45*	0.471	– 0.45	1.2	0.49	0.592	4.43 × 10–3	0.0109
Cumulative COVID-19 cases	– 0.013	0.0161	0.0399	0.0417	5.85 × 10–3	0.0203	– 1.25 × 10–4	3.72 × 10–4
Cumulative COVID-19 deaths	0.118	0.0712	– 0.248	0.184	– 0.0431	0.0897	9.33 × 10–4	1.65 × 10–3
*Worry about being infected with COVID-19*								
(1 = never thought about it, 5 = all the time)	0.0331	0.823	0.101	2.12	– 0.827	1.03	– 0.0158	0.0189
*Subjective risk of COVID-19 infection*								
(1 = highly unlikely, 5 = highly likely)	– 0.847	0.862	7.71*	2.16	1.98	1.07	– 0.0578*	0.0198
Worry × subjective risk	0.206	0.351	– 2.12*	0.9	– 0.0427	0.438	0.0193*	8.08 × 10–3

Note. *N* = 2599, *s.e.* standard error, *significant at 5% level

**Table 7:** Tobit regression results including whether COVID-19 affected health or quality of life

	11111	s.e	Dead	s.e	55555	s.e	55555 rescaled	s.e
Constant	95.4*	6.13	-5.9	15.1	26.8*	7.69	0.148	0.136
Age	0.0483	0.218	0.234	0.551	– 0.0114	0.276	1.53 × 10–3	4.87 × 10–3
Age^2^	– 1.16 × 10–3	2.33 × 10–3	– 5.26 × 10–3	6.02 × 10–3	– 2.12 × 10–3	2.97 × 10–3	– 1.98 × 10–5	5.21 × 10–5
Female	2.98*	1.07	– 2.77	2.69	– 3.6*	1.35	– 2.92 × 10–3	0.0239
White	2.94	1.75	– 12.2*	4.14	– 3.02	2.2	0.057	0.0393
*Education*								
Left school after minimum age	1.56	1.5	– 5.77	3.85	– 2.27	1.91	– 8.12 × 10–3	0.0336
Degree	– 1.31	1.29	4.54	3.3	2.2	1.63	0.0238	0.0287
Live alone	1.36	1.34	– 3.99	3.47	– 3.16	1.71	– 0.0107	0.0299
*Occupation*								
Retired	1.03	2.19	4.71	5.63	0.633	2.79	– 0.0329	0.0491
Employed—key worker	– 1.64	1.64	– 5.31	4.07	– 1.07	2.06	9.54 × 10–4	0.0365
Employed—non-key worker	– 1.65	1.75	4.78	4.24	1.54	2.19	– 0.019	0.039
*EQ-5D*								
Mobility levels 2–5	0.643	1.83	– 4.95	4.64	3.01	2.33	0.0979*	0.0413
Self-care levels 2–5	0.865	2.32	4.57	5.71	2.95	2.95	4.35 × 10–3	0.0528
Usual activities levels 2–5	-1.86	1.93	3.23	4.84	1.55	2.47	– 0.0338	0.0439
Pain/discomfort levels 2–5	0.333	1.28	3.12	3.22	– 0.304	1.63	– 0.0189	0.0287
Anxiety/depression levels 2–5	– 2.09	1.19	7.19*	3	1.73	1.5	– 8.14 × 10–3	0.0266
Number of co-morbidities	0.528	0.562	1.55	1.39	– 7.36 × 10–3	0.715	– 0.0238	0.0127
Description of own health	– 3.96*	0.638	0.77	1.58	0.585	0.805	2.76 × 10–3	0.0143
Cumulative COVID-19 cases	– 8.01 × 10–3	0.0198	0.0318	0.0498	– 4.29 × 10–4	0.0251	– 3.43 × 10–4	– 3.77 × 10–4
Cumulative COVID-19 deaths	0.12	0.0946	– 0.0901	0.239	– 0.0342	0.12	5.88 × 10–4	7 × 10–4
Worry about being infected with COVID-19								
(1 = never thought about it, 5 = all the time)	1.37	1.12	– 2.19	2.81	– 1.57	1.42	– 0.0198	0.0251
Subjective risk of COVID-19 infection								
(1 = highly unlikely, 5 = highly likely)	0.0996	1.15	3.97	2.77	0.537	1.45	– 0.0543*	0.0258
Worry x subjective risk	– 0.223	0.473	– 1.06	1.17	0.0805	0.596	0.0167	0.0106
COVID-19 affected health								
Negatively (baseline no)	– 0.154	1.5	4.67	3.71	4.74*	1.89	0.0168	0.0337
Positively	1.33	2.55	14.8*	5.84	6.18*	3.15	– 0.0336	0.0566
COVID-19 affected quality of life (baseline no)								
Negatively	0.304	1.15	– 2.96	2.94	– 0.993	1.46	– 9.71 × 10–3	0.0258
Positively	– .54	2.09	8.5	5	0.804	2.64	– 0.0763	0.0471

Note. *N* = 1294, *s.e.* standard error, *significant at 5% level

**Table 8:** Tobit regression results including frequency of leaving the house for shopping

	11111	s.e	Dead	s.e	55555	s.e	55555 rescaled	s.e
Constant	107*	4.97	8.27	12.1	21.3*	6.31	– 0.0755	0.109
Age	– 0.164	0.201	– 0.136	0.508	0.0236	0.258	5.34 × 10–3	4.43 × 10–3
Age^2^	1.1 × 10–3	2.15 × 10–3	– 3.23 × 10–3	5.54 × 10–3	– 2.92 × 10–3	2.78 × 10–3	– 4.94 × 10–5	4.75 × 10–5
Female	2.43*	0.985	– 3.88	2.49	– 2.68*	1.27	2.92 × 10–3	0.0218
White	2.36	1.66	– 10.4*	3.95	– 2.01	2.13	0.0448	0.0368
Education								
Left school after minimum age	1.17	1.38	– 5.8	3.56	– 2.25	1.79	– 0.0138	0.0306
Degree	– 0.973	1.17	5.66	3	2.59	1.5	0.0146	0.0257
Live alone	0.869	1.22	– 3.12	3.14	– 3.03	1.58	– 0.013	0.0269
Occupation								
Retired	0.114	1.98	7.61	5.1	2.96	2.57	– 0.0445	0.0439
Employed—key worker	– 1.54	1.47	– 1.12	3.68	0.729	1.89	– 0.0164	0.0325
Employed—non-key worker	– 1.04	1.58	6.13	3.88	2.31	2.02	– 0.0349	0.0349
EQ-5D								
Mobility levels 2–5	– 0.716	1.64	– 5.03	4.22	2.97	2.14	0.0993*	0.0368
Self-care levels 2–5	1.38	2.09	6.94	5.13	4.37	2.71	– 0.0189	0.047
Usual activities levels 2–5	– 2.59	1.73	5.12	4.36	2.81	2.25	– 0.0198	0.0389
Pain/discomfort levels 2–5	0.228	1.15	2.89	2.91	– 1.4	1.5	– 0.036	0.0256
Anxiety/depression levels 2–5	– 1.59	1.05	6.55*	2.66	1.91	1.36	– 7.3 × 10–3	0.0233
Number of co-morbidities	0.909	0.517	1.78	1.28	– 0.38	0.67	– 0.0347*	0.0115
Description of own health	– 3.38*	0.577	– 0.764	1.44	0.695	0.743	0.0153	0.0128
How often go shopping (baseline never)								
< weekly	– 1.56	1.67	1	4.22	– 1.22	2.15	– 0.0111	0.0368
Weekly	– 0.882	1.47	– 2.92	3.76	0.0746	1.89	3.53 × 10–3	0.0324
2–6 times per week	– 1.9	1.67	6.9	4.2	– 0.227	2.16	– 0.0415	0.0369
Daily	– 5.04	2.68	10.4	6.58	5.03	3.49	0.0116	0.0604
> daily	– 0.844	7.05	5.83	17	15.7	8.89	0.201	0.157

Note. *N*  1294, *s.e.* standard error, *significant at 5% level

**Table 9:** Tobit regression results including frequency of leaving the house for exercise and fresh air

	11111	s.e	Dead	s.e	55555	s.e	55555 rescaled	s.e
Constant	108*	4.97	2.45	12.2	17.6*	6.32	– 0.0745	0.109
Age	– 0.164	0.199	– 0.198	0.501	0.0397	0.256	5.34 × 10–3	4.4 × 10–3
Age^2^	9.56 × 10–4	2.12 × 10–3	– 2.08 × 10–3	5.46 × 10–3	– 2.89 × 10–3	2.76 × 10–3	– 5.02 × 10–5	4.72 × 10–5
Female	2.66*	0.976	– 4.52	2.47	– 2.84*	1.26	2.8 × 10–3	0.0217
White	2.01	1.68	– 8.67*	4	– 1.66	2.16	0.0379	0.0375
Education								
Left school after minimum age	1.34	1.38	– 5.52	3.55	– 2.39	1.79	– 0.0175	0.0306
Degree	– 1.04	1.16	5.35	2.99	2.48	1.5	0.0153	0.0257
Live alone	0.686	1.21	– 2.61	3.13	– 2.75	1.58	– 0.0123	0.0269
Occupation								
Retired	0.2	1.97	6.55	5.09	2.65	2.57	– 0.0451	0.044
Employed—key worker	– 1.51	1.47	– 1.59	3.67	0.364	1.89	– 0.018	0.0326
Employed—non-key worker	– 1.12	1.58	6.04	3.86	2.24	2.02	– 0.0352	0.0349
EQ-5D								
Mobility levels 2–5	– 0.893	1.64	– 4.81	4.2	3.15	2.14	0.101*	0.0368
Self-care levels 2–5	1.93	2.08	6.39	5.11	4.22	2.7	– 0.0147	0.047
Usual activities levels 2–5	– 2.57	1.72	4.6	4.32	2.69	2.25	– 0.0196	0.0388
Pain/discomfort levels 2–5	0.113	1.15	2.93	2.91	– 1.33	1.5	– 0.0367	0.0257
Anxiety/depression levels 2–5	– 1.67	1.05	6.86*	2.66	1.85	1.36	– 9.67 × 10–3	0.0234
Number of co-morbidities	0.859	0.514	2.06	1.27	– 0.314	0.668	– 0.0357*	0.0115
Description of own health	– 3.3*	0.578	– 0.814	1.44	0.698	0.745	0.0168	0.0128
How often exercise (baseline never)								
< weekly	– 4.29*	2.02	12.2*	5.04	3.89	2.61	– 0.0155	0.0449
Weekly	– 4.65*	1.98	14.1*	4.96	7.22*	2.56	– 0.0251	0.0442
2–6 times per week	– 1.68	1.69	1.68	4.37	2.59	2.18	0.0255	0.0374
Daily	– 2.15	1.69	5.28	4.36	2.78	2.19	– 5.47 × 10–3	0.0373
> daily	2.59	2.59	6.67	6.5	0.531	3.31	– 0.0131	0.0563

Note. *N*  1294, *s.e.* standard error, *significant at 5% level

### Appendix D Alternative methods of censoring rescaled 55555 values

The main analysis presents results with rescaled 55555 values censored at  – 1. For robustness, additional analyses were run with the censoring threshold of  – 1 and  – 2. In addition, a number of additional analyses were run using four separate criteria for removing participants.

1. Low rescaled 55555 values
  Individuals’ VAS responses were removed from the data if their rescaled 55555 values fell below a given threshold. Analyses were run with thresholds of  – 1,  – 1.5 and  – 2.
2. Large impact on mean rescaled 55555 values
  Similar to the approach used in Webb et al. (2020), individuals were removed from the data if their responses had a large impact on the mean rescaled value of 55555. The influence of each individual on the mean was found by calculating the differences between the mean with or without a given individual included. Individuals were then excluded if their influence was more than a given number of standard deviations away from the mean influence. Analyses were run with thresholds of 1.5, 2 and 2.5 standard deviations.
3. High VAS rating for dead
  Many extremely low rescaled values are as a result of an arguably implausibly high rating for dead. Thus, analyses were re-run excluding respondents who rated dead above thresholds of 75, 50 and 25.
4. High rate of change of rescaled 55555 values with respect to raw values
  A potential cause of extremely low rescaled 55555 values was placing 11111 and dead very close together, meaning that small changes to 55555 on the 100–0 scale led to large changes on the 1–0 scale. Such rescaled values were unlikely to be accurate, so participants were excluded if their rate of change was above a given threshold. The rate of change of the rescaled value of 55555 with respect to the raw value is
  $${\raise0.7ex\hbox{${\partial VAS_{55555}^{{{\text{rescaled}}}} }$} \!\mathord{\left/ {\vphantom {{\partial VAS_{55555}^{{{\text{rescaled}}}} } {\partial VAS_{55555} }}}\right.\kern-0pt} \!\lower0.7ex\hbox{${\partial VAS_{55555} }$}} = {\raise0.7ex\hbox{$1$} \!\mathord{\left/ {\vphantom {1 {\left( {VAS_{11111} - VAS_{{{\text{dead}}}} } \right)}}}\right.\kern-0pt} \!\lower0.7ex\hbox{${\left( {VAS_{11111} - VAS_{{{\text{dead}}}} } \right)}$}}$$
  Participants were excluded from the analysis if $\partial VAS_{55555}^{rescaed} /\partial VAS_{55555}$ exceeded a given threshold, i.e. if changing the rating of 55555 by 1 on the 100–0 scale implied a change of more than the threshold on the full health = 1, dead = 0 scale. Analyses were run with thresholds of 0.1, 0.075 and 0.05.

Table 10 gives the mean VAS responses for the various exclusion criteria. Tables 11, 12, 13 and 14 give the COVID-19-related coefficients for all robustness analyses. Full results including control variables are available upon request to the corresponding author.

See below Appendix D Tables 10, 11, 12, 13 and 14 here.

**Table 10:** Mean VAS responses with different exclusion criteria

Exclusion criteria	Threshold	11111	Dead	55555	55555 rescaled	*N*
Low rescaled 55555	– 1	90.5	6.17	17.4	0.122	2450
		(64.9, 116)	( – 21.8, 34.1)	( – 20.3, 55.1)	( – 0.439, 0.684)	
	– 1.5	90.5	7.11	17.4	0.0973	2499
		(65.0, 116)	( – 23.6, 37.9)	( – 20.4, 55.1)	( – 0.559, 0.753)	
	– 2	90.5	7.73	17.4	0.0762	2528
		(65.0, 116)	( – 24.9, 40.4)	( – 20.4, 55.1)	( – 0.681, 0.834)	
Censoring rescaled 55555	– 1	90.4	9.6	17.4	0.058	2599
		(64.7, 116)	( – 29.7, 48.9)	( – 20.4, 55.3)	( – 0.689, 0.805)	
	– 1.5	90.4	9.6	17.4	0.058	2599
		(64.7, 116)	( – 29.7, 48.9)	( – 20.4, 55.3)	( – 0.689, 0.805)	
	– 2	90.4	9.6	17.4	0.058	2599
		(64.7, 116)	( – 29.7, 48.9)	( – 20.4, 55.3)	( – 0.689, 0.805)	
Impact on mean rescaled 55555	1.5	90.3	9.2	17.5	– 0.0752	2587
		(64.6, 116)	( – 28.4, 46.9)	( – 20.3, 55.2)	( – 3.02, 2.87)	
	2	90.3	9.27	17.4	– 0.105	2589
		(64.6, 116)	( – 28.7, 47.2)	( – 20.3, 55.2)	( – 3.71, 3.50)	
	2.5	90.3	9.3	17.4	– 0.125	2590
		(64.6, 116)	( – 28.8, 47.4)	( – 20.3, 55.2)	( – 4.25, 4.00)	
High VAS for dead	75	90.3	8.02	17.1	0.025	2547
		(64.4, 116)	( – 25.0, 41.1)	( – 19.7, 53.9)	( – 1.50, 1.55)	
	50	90.3	5.14	16.2	0.098	2412
		(64.3, 116)	( – 18.1, 28.4)	( – 18.5, 50.8)	( – 1.05, 1.24)	
	25	90.7	1.79	15.4	0.148	2198
		(64.8, 117)	( – 7.25, 10.8)	( – 17.7, 48.5)	( – 0.907, 1.20)	
High rate of change of rescaled 55555	0.1	90.8	8.2	17.1	0.0337	2539
		(66.9, 115)	( – 26.0, 42.4)	( – 19.8, 53.9)	( – 1.09, 1.16)	
	0.075	90.9	7.84	17	0.052	2520
		(67.8, 114)	( – 25.3, 41.0)	( – 19.9, 53.8)	( – 0.906, 1.01)	
	0.05	91.2	7.26	16.9	0.0748	2490
		(68.8, 113)	( – 23.9, 38.5)	( – 19.9, 53.7)	( – 0.723, 0.873)	

*Note.* 95% confidence intervals in parentheses

**Table 11:** Rescaled 55555 robustness tests for Tobit regressions

		Low rescaled 55555			Censoring rescaled 55555			Impact on mean rescaled 55555			High VAS for dead			Rescaled 55555 high rate of change		
		– 1	– 1.5	– 2	– 1	– 1.5	– 2	– 1	– 1.5	– 2.5	75	50	25	0.1	0.075	0.05
11111	Cases	– 0.0136	– .0126	– 0.0137	– 0.013	– 0.013	– 0.013	– 0.0147	– 0.0147	– 0.0147	– 0.0158	– 0.0166	– 0.0168	– 0.0198	– 0.0138	– 0.0126
		(0.0166)	(0.0163)	(0.0162)	(0.0161)	(0.0161)	(0.0161)	(0.0162)	(0.0162)	(0.0161)	(0.0163)	(0.0169)	(0.0175)	(0.0153)	(0.0151)	(0.0148)
	Deaths	0.116	0.112	0.118	0.118	0.118	0.118	0.128	0.127	0.127	0.136	0.131	0.129	0.142	0.113	0.105
		(0.0732)	(0.0719)	(0.0715)	(0.0712)	(0.0712)	(0.0712)	(0.0714)	(0.0714)	(0.0714)	(0.0721)	(0.0749)	(0.0773)	(0.0675)	(0.0667)	(0.0651)
	Worry	– 0.0152	– 0.108	0.0671	0.0331	0.0331	0.0331	0.0691	0.0633	0.0619	0.118	– 0.24	– 0.102	– 0.0553	– 0.221	– 0.2
		(0.867)	(0.85)	(0.834)	(0.823)	(0.823)	(0.823)	(0.824)	(0.824)	(0.823)	(0.842)	(0.883)	(0.951)	(0.789)	(0.782)	(0.769)
	Risk	– 0.224	– 0.474	– 0.429	– 0.847	– 0.847	– 0.847	– 0.921	– 0.892	– 0.892	– 0.714	– 0.448	– 0.516	– 0.846	– 1.03	– 1.04
		(0.922)	(0.908)	(0.888)	(0.862)	(0.862)	(0.862)	(0.865)	(0.864)	(0.864)	(0.889)	(0.949)	(1.04)	(0.844)	(0.836)	(0.823)
	Worry x risk	0.132	0.205	0.158	0.206	0.206	0.206	0.223	0.215	0.215	0.168	0.214	0.177	0.206*	0.273	0.227
		(0.375)	(0.369)	(0.36)	(0.351)	(0.351)	(0.351)	(0.352)	(0.352)	(0.352)	(0.362)	(0.384)	(0.413)	(0.341)	(0.337)	(0.332)
Dead	Cases	– 8.97 × 10–4	-3.4 × 10–3	– 3.07 × 10–4	0.0399	0.0399	0.0399	0.0239	0.0217	0.0235	8.77 × 10–3	0.0144	5.62 × 10–3	0.0208	0.0137	5.1 × 10–3
		(0.0334)	(0.035)	(0.0367)	(0.0417)	(0.0417)	(0.0417)	(0.0401)	(0.0404)	(0.0405)	(0.0366)	(0.0292)	(0.0144)	(0.0381)	(0.0376)	(0.0363)
	Deaths	– 0.0556	– 0.0417	– 0.051	– 0.248	– 0.248	– 0.248	– 0.159	– 0.155	– 0.162	– 0.0889	– 0.117	– 0.0223	– 0.147	– 0.116	– 0.0777
		(0.147)	(0.154)	(0.162)	(0.184)	(0.184)	(0.184)	(0.177)	(0.178)	(0.179)	(0.161)	(0.129)	(0.0634)	(0.168)	(0.166)	(0.16)
	Worry	– 0.693	1.24	0.247	0.101	0.101	0.101	0.5	0.513	0.348	– 0.311	– 0.542	– 0.211	0.5	0.915	0.57
		(1.74)	(1.82)	(1.88)	(2.12)	(2.12)	(2.12)	(2.02)	(2.04)	(2.05)	(1.87)	(1.52)	(0.768)	(1.95)	(1.92)	(1.87)
	Risk	4.65*	6.03*	5.73*	7.71*	7.71*	7.71*	6.72*	7.17*	7.17*	5.25*	3.09	0.567	4.8*	5.07*	4.93*
		(1.79)	(1.9)	(1.96)	(2.16)	(2.16)	(2.16)	(2.08)	(2.09)	(2.1)	(1.93)	(1.58)	(0.825)	(2.05)	(2.02)	(1.96)
	Worry x risk	– 1.44	– 1.97*	– 1.57	– 2.12*	– 2.12*	– 2.12*	– 1.91*	– 2.06*	– 1.97*	– 1.48	– 1.29	-0.192	-1.54	-1.73*	-1.52
		(0.751)	(0.79)	(0.807)	(0.9)	(0.9)	(0.9)	(0.862)	(0.869)	(0.871)	(0.802)	(0.662)	(0.334)	(0.84)	(0.829)	(0.805)
55555	Cases	6.05 × 10–3	5.09 × 10–3	2.38 × 10–3	5.85 × 10–3	5.85 × 10–3	5.85 × 10–3	7.74 × 10–3	8.29 × 10–3	8.39 × 10–3	4.49 × 10–3	5.65 × 10–3	2.52 × 10–3	7.5 × 10–3	5.33 × 10–3	7.29 × 10–3
		(0.0208)	(0.0207)	(0.0206)	(0.0203)	(0.0203)	(0.0203)	(0.0203)	(0.0203)	(0.0203)	(0.0201)	(0.0196)	(0.0197)	(0.0201)	(0.0203)	(0.0204)
	Deaths	– 0.0451	– 0.0401	– 0.0284	– 0.0431	– 0.0431	– 0.0431	– 0.0479	– 0.0496	-5 × 10–2	-0.0281	-0.0277	-0.0165	-0.0444	-0.036	-0.0444
		(0.0919)	(0.0912)	(0.091)	(0.0897)	(0.0897)	(0.0897)	(0.0898)	(0.0898)	(0.0898)	(0.0888)	(0.0869)	(0.0869)	(0.0889)	(0.0894)	(0.0901)
	Worry	– 0.585	– 0.758	– 0.729	– 0.827	– 0.827	– 0.827	– 0.844	– 0.853	– 0.863	– 0.843	– 0.714	-0.934	-0.736	-0.642	-0.596
		(1.08)	(1.07)	(1.06)	(1.03)	(1.03)	(1.03)	(1.03)	(1.03)	(1.03)	(1.03)	(1.02)	(1.06)	(1.03)	(1.04)	(1.06)
	Risk	2.2	2.04	2.11	1.98	1.98	1.98	2.04	1.95	1.95	1.76	1.03	1.23	1.67	1.74	1.72
		(1.14)	(1.14)	(1.12)	(1.07)	(1.07)	(1.07)	(1.07)	(1.07)	(1.07)	(1.08)	(1.09)	(1.15)	(1.1)	(1.11)	(1.12)
	Worry x risk	– 0.171	– 0.0963	– 0.0855	– .0427	– 0.0427	– 0.0427	– 0.0774	– 0.0502	– 0.0448	– 0.0345	0.0536	0.0614	-0.0151	-0.0411	-0.0714
		(0.467)	(0.464)	(0.454)	(0.438)	(0.438)	(0.438)	(0.439)	(0.438)	(0.438)	(0.442)	(0.441)	(0.459)	(0.445)	(0.447)	(0.454)
55555 rescaled	Cases	2.48 × 10–4	2.51 × 10–4*	1.78 × 10–4	-1.25 × 10–4*	– 1.25 × 10–4*	– 1.25 × 10–4*	– 1.85 × 10–3*	– 1.35 × 10–3*	– 1.87 × 10–3*	– 1.66 × 10–4	5.49 × 10–5	2.5 × 10–4	-2.37 × 10–4	-1.64 × 10–4	2.44 × 10–4
		(2.69 × 10–4)	(3.12 × 10–4)	(3.65 × 10–4)	(3.72 × 10–4)	(3.72 × 10–4)	(3.72 × 10–4)	(1.39 × 10–3)	(1.78 × 10–3)	(2.08 × 10–3)	(7.67 × 10–4)	(5.9 × 10–4)	(5.74 × 10–4)	(5.26 × 10–4)	(4.69 × 10–4)	(3.84 × 10–4)
	Deaths	– 8.67 × 10–4	– 9.04 × 10–4	– 6.4 × 10–4	9.33 × 10–4	9.33 × 10–4	9.33 × 10–4	8.35 × 10–3	7.68 × 10–3	9.61 × 10–3	2.44 × 10–3	1.11 × 10–3	-3.26 × 10–5	1.59 × 10–3	1.26 × 10–3	-5.3 × 10–4
		(1.19 × 10–3)	(1.38 × 10–3)	(1.61 × 10–3)	(1.65 × 10–3)	(1.65 × 10–3)	(1.65 × 10–3)	(6.13 × 10–3)	(7.86 × 10–3)	(9.19 × 10–3)	(3.39 × 10–3)	(2.62 × 10–3)	(2.53 × 10–3)	(2.32 × 10–3)	(2.07 × 10–3)	(1.69 × 10–3)
	Worry	9.51 × 10–4	– 0.0266	– 9.39 × 10–3	– 0.0158	– 0.0158	– 0.0158	– 0.126	– 0.128	– 0.0714	0.0104	0.0164	9.01 × 10–3	-0.0173	-0.0212	-4.21 × 10–3
		(0.014)	(0.0162)	(0.0188)	(0.0189)	(0.0189)	(0.0189)	(0.0705)	(0.0906)	(0.106)	(0.0395)	(0.0307)	(0.0309)	(0.027)	(0.0241)	(0.0198)
	Risk	– 0.0138	– 0.0363*	– 0.0328	– 0.0578*	– 0.0578*	– 0.0578*	– 0.174*	– 0.29*	– 0.289*	– 0.0733	– 1.94 × 10–3	0.0346	-0.028	-0.0301*	-0.023
		(0.0149)	(0.0173)	(0.0199)	(0.0198)	(0.0198)	(0.0198)	(0.0739)	(0.0944)	(0.11)	(0.0415)	(0.0328)	(0.0335)	(0.0288)	(0.0257)	(0.0212)
	Worry x risk	8.83 × 10–3	0.0171*	0.0114	0.0193*	0.0193*	0.0193*	0.0652*	0.101*	0.0695	0.0104	– 3.56 × 10–3	-0.016	0.0133	0.0176	8.34 × 10–3
		(6.07 × 10–3)	(7.04 × 10–3)	(8.08 × 10–3)	(8.08 × 10–3)	(8.08 × 10–3)	(8.08 × 10–3)	(0.0302)	(0.0387)	(0.0451)	(0.017)	(0.0133)	(0.0134)	(0.0117)	(0.0104)	(8.57 × 10–3)

*Note.* Standard errors in parentheses. *significant at 5% level

**Table 12:** Rescaled 55555 robustness tests for Tobit regression results including whether COVID-19 affected health or quality of life

		Low rescaled 55555			Censoring rescaled 55555			Impact on mean rescaled 55555			High VAS for dead			Rescaled 55555 high rate of change		
		– 1	– 1.5	– 2	– 1	– 1.5	– 2	1.5	2	2.5	75	50	25	0.1	0.075	0.05
11111	Cases	– 7.59 × 10–3	– 7.8 × 10–3	– 9.16 × 10–3	– 8.01 × 10–3	– 8.01 × 10–3	– 8.01 × 10–3	– 8.87 × 10–3	– 8.87 × 10–3	– 8.82 × 10–3	– 8.81 × 10–3	-0.0122	-9.54 × 10–3	-0.0114	-6.38 × 10–3	-7.44 × 10–3
		(0.0201)	(0.0198)	(0.0197)	(0.0198)	(0.0198)	(0.0198)	(0.0198)	(0.0198)	(0.0198)	(2 × 10–2)	(0.0206)	(0.0212)	(0.019)	(0.0189)	(0.0183)
	Deaths	0.109	0.105	0.11	0.12	0.12	0.12	0.126	0.126	0.126	0.127	0.132	0.111	0.123	0.0989	0.092
		(0.0958)	(0.0943)	(0.094)	(0.0946)	(0.0946)	(0.0946)	(0.0947)	(0.0947)	(0.0946)	(0.0956)	(0.0986)	(0.101)	(0.0905)	(0.0902)	(0.0873)
	Worry	1.38	1.12	1.32	1.37	1.37	1.37	1.43	1.43	1.42	1.41	1.17	1.83	0.635	0.203	0.248
		(1.16)	(1.13)	(1.11)	(1.12)	(1.12)	(1.12)	(1.12)	(1.12)	(1.12)	(1.13)	(1.19)	(1.26)	(1.09)	(1.09)	(1.05)
	Risk	0.412	0.227	0.296	0.0996	0.0996	0.0996	0.112	0.112	0.112	0.133	0.129	0.324	-0.525	-0.875	-0.612
		(1.2)	(1.18)	(1.16)	(1.15)	(1.15)	(1.15)	(1.16)	(1.16)	(1.15)	(1.17)	(1.24)	(1.34)	(1.13)	(1.14)	(1.1)
	Worry x risk	– 0.209	– 0.105	– 0.19	– 0.223	– 0.223	– 0.223	– 0.229	– 0.229	-0.226	-0.225	-0.146	-0.333	0.0427	0.182	0.0717
		(0.495)	(0.486)	(0.474)	(0.473)	(0.473)	(0.473)	(0.474)	(0.474)	(0.473)	(0.478)	(0.509)	(0.542)	(0.461)	(0.461)	(0.446)
	C19 health negative	0.409	0.204	– 5.81 × 10–3	– 0.154	– 0.154	– 0.154	– 0.165	– 0.165	– 0.154	– 0.0624	– 0.133	0.686	-0.0867	-0.119	0.0975
		(1.55)	(1.52)	(1.5)	(1.5)	(1.5)	(1.5)	(1.5)	(1.5)	(1.5)	(1.52)	(1.58)	(1.64)	(1.44)	(1.43)	(1.39)
	C19 health positive	2.31	1.77	1.83	1.33	1.33	1.33	1.41	1.41	1.41	1.84	2.91	2.4	1.57	1.63	1.74
		(2.69)	(2.6)	(2.58)	(2.55)	(2.55)	(2.55)	(2.56)	(2.56)	(2.56)	(2.62)	(2.88)	(3.13)	(2.47)	(2.48)	(2.43)
	C19 QOL negative	0.552	0.697	0.601	0.304	0.304	0.304	0.344	0.344	0.346	0.348	0.363	-0.246	0.25	0.0583	0.14
		(1.17)	(1.16)	(1.15)	(1.15)	(1.15)	(1.15)	(1.15)	(1.15)	(1.15)	(1.17)	(1.2)	(1.23)	(1.1)	(1.1)	(1.06)
	C19 QOL positive	– 3.21	– 3.35	– 3.41	– 3.54	– 3.54	– 3.54	– 3.63	– 3.63	– 3.64	– 3.79	– 3.34	-4.08	-3.44	-3.61	-2.32
		(2.21)	(2.14)	(2.13)	(2.09)	(2.09)	(2.09)	(2.11)	(2.11)	(2.11)	(2.15)	(2.31)	(2.4)	(2.04)	(2.04)	(2.01)
Dead	Cases	– 7.83 × 10–3	– 8.59 × 10–3	9.2 × 10–3	0.0318	0.0318	0.0318	0.0172	0.0172	0.0213	1.47 × 10–3	0.0264	-5.2 × 10–5	0.0278	4.88 × 10–3	1.25 × 10–4
		(0.039)	(0.0411)	(0.0431)	(0.0498)	(0.0498)	(0.0498)	(0.0473)	(0.0473)	(0.0477)	(0.0434)	(0.0342)	(0.0179)	(0.045)	(0.0438)	(0.0428)
	Deaths	0.0669	0.0981	0.0451	– 0.0901	– 0.0901	– 0.0901	– 0.0105	– 0.0105	– 0.0335	0.0845	– 0.0841	0.016	-1.93 × 10–3	0.103	0.0944
		(0.187)	(0.197)	(0.207)	(0.239)	(0.239)	(0.239)	(0.227)	(0.227)	(0.229)	(0.208)	(0.165)	(0.0855)	(0.216)	(0.21)	(0.205)
	Worry	– 3.97	– 1.3	– 1.81	– 2.19	– 2.19	– 2.19	– 1.45	– 1.45	– 1.82	– 2.17	– 2.03	-0.128	-1.1	-0.652	-1.07
		(2.26)	(2.35)	(2.43)	(2.81)	(2.81)	(2.81)	(2.66)	(2.66)	(2.69)	(2.45)	(1.98)	(1.06)	(2.56)	(2.51)	(2.45)
	Risk	2.02	2.95	3.12	3.97	3.97	3.97	4.12	4.12	4.13	4.02	2.24	1.46	3.58	4.11	3.87
		(2.2)	(2.36)	(2.44)	(2.77)	(2.77)	(2.77)	(2.64)	(2.64)	(2.66)	(2.43)	(1.98)	(1.09)	(2.58)	(2.53)	(2.47)
	Worry x risk	– 0.454	– 1.14	– 0.987	– 1.06	– 1.06	– 1.06	– 1.2	– 1.2	– 1.03	– 0.95	– 0.939	-0.555	-1.08	-1.25	-1.07
		(0.945)	(0.995)	(1.02)	(1.17)	(1.17)	(1.17)	(1.11)	(1.11)	(1.11)	(1.02)	(0.842)	(0.454)	(1.07)	(1.05)	(1.02)
	C19 health negative	2.53	3.2	3.26	4.67	4.67	4.67	3.67	3.67	4.42	3.26	2.16	0.977	3.92	2.21	1.95
		(2.97)	(3.12)	(3.25)	(3.71)	(3.71)	(3.71)	(3.52)	(3.52)	(3.54)	(3.25)	(2.59)	(1.35)	(3.36)	(3.29)	(3.22)
	C19 health positive	14.9*	16.5*	15*	14.8*	14.8*	14.8*	15.4*	15.4*	15.3*	14.7*	8.37	-0.586	15.2*	13.6*	13.7*
		(4.69)	(4.85)	(5.12)	(5.84)	(5.84)	(5.84)	(5.52)	(5.52)	(5.58)	(5.14)	(4.39)	(2.52)	(5.31)	(5.21)	(5.15)
	C19 QOL negative	– 1.27	– 2.97	– 3.37	– 2.96	– 2.96	– 2.96	– 2.21	– 2.21	-2.12	-2.46	-0.484	-0.115	-2.44	-2.21	-2.02
		(2.31)	(2.44)	(2.55)	(2.94)	(2.94)	(2.94)	(2.79)	(2.79)	(2.82)	(2.57)	(2.02)	(1.05)	(2.66)	(2.58)	(2.51)
	C19 QOL positive	4.34	5.57	5.29	8.5	8.5	8.5	7.51	7.51	7.39	6.08	1.53	1.83	5.85	5.72	3.52
		(4.13)	(4.24)	(4.44)	(5)	(5)	(5)	(4.78)	(4.78)	(4.83)	(4.44)	(3.78)	(1.96)	(4.63)	(4.51)	(4.5)
55555	Cases	5.61 × 10–3	3.46 × 10–3	3.67 × 10–4	-4.29 × 10–4	-4.29 × 10–4	-4.29 × 10–4	1.01 × 10–3	1.01 × 10–3	1.14 × 10–3	-8.3 × 10–4	6.36 × 10–3	2.65 × 10–3	3.73 × 10–3	-6.62 × 10–4	4.81 × 10–5
		(0.0256)	(0.0253)	(0.0253)	(0.0251)	(0.0251)	(0.0251)	(0.0251)	(0.0251)	(0.025)	(0.0249)	(0.0245)	(0.0247)	(0.0249)	(0.0251)	(0.0251)
	Deaths	– 0.0604	– 0.0588	– 0.0451	– 0.0342	– 0.0342	– 0.0342	– 0.0409	– 0.0409	-0.0417	-0.0246	-0.063	-0.0224	-0.0446	-0.0242	-0.0318
		(0.122)	(0.121)	(0.121)	(0.12)	(0.12)	(0.12)	(0.12)	(0.12)	(0.12)	(0.119)	(0.118)	(0.118)	(0.119)	(0.12)	(0.12)
	Worry	– 1.01	– 1.29	– 1.56	– 1.57	– 1.57	– 1.57	– 1.61	– 1.61	-1.62	-1.73	-1.04	-0.0528	-1.56	-1.47	-1.44
		(1.48)	(1.45)	(1.43)	(1.42)	(1.42)	(1.42)	(1.42)	(1.42)	(1.41)	(1.41)	(1.41)	(1.46)	(1.42)	(1.43)	(1.43)
	Risk	1.04	0.863	0.668	0.537	0.537	0.537	0.485	0.485	0.484	0.485	0.624	2.39	0.599	0.634	0.645
		(1.51)	(1.5)	(1.47)	(1.45)	(1.45)	(1.45)	(1.44)	(1.44)	(1.44)	(1.44)	(1.46)	(1.54)	(1.47)	(1.48)	(1.49)
	Worry x risk	– 0.177	– 0.0605	0.0371	0.0805	0.0805	0.0805	0.0776	0.0776	0.0849	0.145	-0.0821	-0.693	0.0834	0.0698	0.0426
		(0.629)	(0.62)	(0.606)	(0.596)	(0.596)	(0.596)	(0.596)	(0.596)	(0.596)	(0.593)	(0.604)	(0.628)	(0.603)	(0.607)	(0.608)
	C19 health negative	4.56*	4.52*	4.47*	4.74*	4.74*	4.74*	4.73*	4.73*	4.76*	4.54*	4.84*	5.15*	4.69*	4.37*	4.51*
		(1.97)	(1.95)	(1.93)	(1.89)	(1.89)	(1.89)	(1.9)	(1.9)	(1.89)	(1.89)	(1.88)	(1.9)	(1.89)	(1.9)	(1.91)
	C19 health positive	6.34	5.93	6.28	6.18*	6.18*	6.18*	6.09	6.09	6.08	6.47*	3.34	0.589	5.75	6.35*	5.67
		(3.32)	(3.24)	(3.23)	(3.15)	(3.15)	(3.15)	(3.16)	(3.16)	(3.15)	(3.18)	(3.33)	(3.54)	(3.17)	(3.2)	(3.24)
	C19 QOL negative	– 1.69	– 1.53	– 1.27	– 0.993	– 0.993	– 0.993	– 1.12	– 1.12	-1.12	-1.02	-1.18	-0.925	-1.05	-0.905	-0.94
		(1.5)	(1.48)	(1.48)	(1.46)	(1.46)	(1.46)	(1.46)	(1.46)	(1.46)	(1.45)	(1.43)	(1.44)	(1.45)	(1.46)	(1.46)
	C19 QOL positive	1.17	0.841	0.942	0.804	0.804	0.804	0.898	0.898	0.892	0.403	0.55	2.06	0.883	0.26	0.151
		(2.81)	(2.74)	(2.72)	(2.64)	(2.64)	(2.64)	(2.66)	(2.66)	(2.65)	(2.67)	(2.74)	(2.78)	(2.67)	(2.69)	(2.74)
55555 rescaled	Cases	4.46 × 10–5	1.02 × 10–5	– 3.27 × 10–4	– 3.77 × 10–4	– 3.77 × 10–4	– 3.77 × 10–4	– 1.31 × 10–3	– 1.31 × 10–3	-2.37 × 10–3	-1.4 × 10–4	-3.83 × 10–4	1.34 × 10–4	-7.13 × 10–4	-2.08 × 10–4	5.66 × 10–6
		(3.31 × 10–4)	(3.76 × 10–4)	(4.34 × 10–4)	(4.43 × 10–4)	(4.43 × 10–4)	(4.43 × 10–4)	(1.62 × 10–3)	(1.62 × 10–3)	(2.51 × 10–3)	(1.08 × 10–3)	(9.43 × 10–4)	(9.81 × 10–4)	(6.13 × 10–4)	(5.22 × 10–4)	(4.56 × 10–4)
	Deaths	– 9.83 × 10–4	– 1.3 × 10–3	– 2.34 × 10–4	7 × 10–4	7 × 10–4	7 × 10–4	1 × 10–2	1 × 10–2	0.0166	4.01 × 10–3	4.1 × 10–3	2.8 × 10–3	1.42 × 10–3	-8.74 × 10–4	-1.14 × 10–3
		(1.58 × 10–3)	(1.79 × 10–3)	(2.07 × 10–3)	(2.12 × 10–3)	(2.12 × 10–3)	(2.12 × 10–3)	(7.74 × 10–3)	(7.74 × 10–3)	(0.012)	(5.15 × 10–3)	(4.53 × 10–3)	(4.68 × 10–3)	(2.93 × 10–3)	(2.49 × 10–3)	(2.18 × 10–3)
	Worry	0.0122	– 0.0255	– 0.022	– 0.0198	– 0.0198	– 0.0198	– 0.0437	– 0.0437	0.0836	-0.0225	0.0262	0.0318	-0.0321	-0.0323	-0.0189
		(0.0193)	(0.0216)	(0.0246)	(0.0251)	(0.0251)	(0.0251)	(0.0921)	(0.0921)	(0.143)	(0.0611)	(0.0546)	(0.0583)	(0.0351)	(0.0299)	(0.0261)
	Risk	– 0.0167	– 0.0332	– 0.0428	– 0.0543	– 0.0543	– 0.0543	– 0.173	– 0.173	-0.169	-0.124	0.0209	0.0688	-0.041	-0.0538	-0.0491
		(0.0197)	(0.0224)	(0.0255)	(0.0258)	(0.0258)	(0.0258)	(0.0941)	(0.0941)	(0.146)	(0.0628)	(0.0568)	(0.0617)	(0.0365)	(0.0312)	(0.0272)
	Worry × risk	4.42 × 10–3	0.0151	0.0155	0.0167	0.0167	0.0167	0.0377	0.0377	-0.0249	0.0222	-0.0217	-0.0401	0.0151	0.0186	0.0136
		(8.19 × 10–3)	(9.26 × 10–3)	(0.0105)	(0.0106)	(0.0106)	(0.0106)	(0.0388)	(0.0388)	(6 × 10–2)	(0.0258)	(0.0234)	(0.0251)	(0.0149)	(0.0127)	(0.0111)
	C19 health negative	0.0435	0.0295	0.0222	0.0168	0.0168	0.0168	0.0571	0.0571	-0.205	0.0587	0.091	0.127	-7.03 × 10–3	0.0405	0.0519
		(0.0257)	(0.0291)	(0.0334)	(0.0337)	(0.0337)	(0.0337)	(0.124)	(0.124)	(0.19)	(0.0824)	(0.073)	(0.0764)	(0.0468)	(0.0399)	(0.035)
	C19 health positive	– 0.0377	– 0.0704	– 0.0303	– 0.0336	– 0.0336	– 0.0336	– 0.118	– 0.118	-0.0876	-0.0494	-8.31 × 10–3	0.0209	-0.0983	0.0178	-9.32 × 10–4
		(0.0437)	(0.0488)	(0.0563)	(0.0566)	(0.0566)	(0.0566)	(0.206)	(0.206)	(0.319)	(0.139)	(0.13)	(0.142)	(0.0789)	(0.0677)	(0.0597)
	C19 QOL negative	– 0.0269	– 2.36 × 10–3	2.59 × 10–3	– 9.71 × 10–3	– 9.71 × 10–3	– 9.71 × 10–3	– 0.0557	– 0.0557	-0.0943	-0.0598	-0.074	-0.0786	-0.012	-4.52 × 10–3	-0.0111
		(0.0193)	(0.022)	(0.0253)	(0.0258)	(0.0258)	(0.0258)	(0.0943)	(0.0943)	(0.146)	(0.0627)	(0.055)	(0.057)	(0.0357)	(0.0303)	(0.0264)
	C19 QOL positive	2.38 × 10–3	– 0.0314	– 0.0313	– 0.0763	– 0.0763	– -0.0763	– 0.157	– 0.157	-0.104	-0.176	0.0507	0.0713	-0.0326	-0.077	-0.0268
		(0.0366)	(0.0409)	(0.047)	(0.0471)	(0.0471)	(0.0471)	(0.172)	(0.172)	(0.267)	(0.116)	(0.106)	(0.112)	(0.0661)	(0.0564)	(5 × 10–2)

*Note.* Standard errors in parentheses; * = significant at 5% level

**Table 13:** Rescaled 55555 robustness tests for Tobit regression results including frequency of leaving the house for shopping

		Low rescaled 55555			Censoring rescaled 55555			Impact on mean rescaled 55555			High VAS for dead			Rescaled 55555 high rate of change		
		– 1	– 1.5	– 2	– 1	– 1.5	– 2	1.5	2	2.5	75	50	25	0.1	0.075	0.05
11111	Shopping < weekly	– 1.48	– 1.5	– 1.38	– 1.56	– 1.56	– 1.56	– 1.54	– 1.54	– 1.54	– 1.55	– 1.15	– 1.53	– 1.32	– 1.33	– 1.23
		(1.7)	(1.68)	(1.67)	(1.67)	(1.67)	(1.67)	(1.67)	(1.67)	(1.67)	(1.69)	(1.75)	(1.79)	(1.62)	(1.6)	(1.53)
	Shopping 2–6 times/week	– 1.78	– 1.95	– 1.81	– 1.9	– 1.9	– -1.9	– 1.86	– 1.86	– 1.85	– 1.84	– 1.73	– 1.97	– 1.46	– 1.61	– 1.99
		(1.71)	(1.68)	(1.67)	(1.67)	(1.67)	(1.67)	(1.68)	(1.68)	(1.68)	(1.7)	(1.75)	(1.81)	(1.62)	(1.61)	(1.54)
	Shopping weekly	– 0.59	– 0.593	– 0.539	– 0.882	– 0.882	– 0.882	– 0.789	– 0.789	– 0.79	– 0.727	– 0.499	– 0.684	– 0.792	– 0.874	– 0.714
		(1.49)	(1.47)	(1.47)	(1.47)	(1.47)	(1.47)	(1.48)	(1.48)	(1.48)	(1.49)	(1.54)	(1.57)	(1.42)	(1.4)	(1.35)
	Shopping daily	– 5.53	– 5.41	– 5.28	– 5.04	– 5.04	– 5.04	– 4.95	– 4.95	– 4.96	– 5.24	– 5.13	– 6.7	– 5.03	– 5.3	– 6.05
		(2.76)	(2.7)	(2.69)	(2.68)	(2.68)	(2.68)	(2.68)	(2.68)	(2.68)	(2.73)	(2.81)	(2.95)	(2.59)	(2.56)	(2.45)
	Shopping > daily	– 0.77	– 0.777	– 0.525	– 0.844	– 0.844	– 0.844	– 0.685	– 0.685	– 0.685	– 0.677	– 0.642	– 5.25	– 0.379	– 0.851	– 1.22
		(7.04)	(6.99)	(6.98)	(7.05)	(7.05)	(7.05)	(7.06)	(7.06)	(7.06)	(7.1)	(7.14)	(8.55)	(6.76)	(6.66)	(6.35)
Dead	Shopping < weekly	0.477	0.302	– 0.631	1	1	1	1.45	1.45	1.4	– 0.331	2.09	0.343	– 0.221	– 0.675	– 1.53
		(3.37)	(3.57)	(3.7)	(4.22)	(4.22)	(4.22)	(4.06)	(4.06)	(4.09)	(3.68)	(2.96)	(1.5)	(3.84)	(3.72)	(3.64)
	Shopping 2–6 times/week	3.79	5.19	4.61	6.9	6.9	6.9	6.15	6.15	6.63	3.75	6.1*	0.56	5.36	4.54	4.18
		(3.39)	(3.54)	(3.67)	(4.2)	(4.2)	(4.2)	(4.05)	(4.05)	(4.08)	(3.67)	(2.93)	(1.52)	(3.81)	(3.7)	(3.6)
	Shopping weekly	– 1.5	– 1.12	– 2.24	– 2.92	– 2.92	– 2.92	– 1.69	– 1.69	– 1.8	– 2.91	0.39	– 0.791	– 2.04	– 1.97	– 2.3
		(2.99)	(3.15)	(3.26)	(3.76)	(3.76)	(3.76)	(3.62)	(3.62)	(3.65)	(3.26)	(2.63)	(1.33)	(3.39)	(3.28)	(3.19)
	Shopping daily	7.12	9.57	7.69	10.4	10.4	10.4	11.1	11.1	11	6.68	9.14*	4.54	9.61	9.81	8.19
		(5.38)	(5.55)	(5.8)	(6.58)	(6.58)	(6.58)	(6.3)	(6.3)	(6.35)	(5.83)	(4.55)	(2.35)	(5.95)	(5.73)	(5.64)
	Shopping > daily	9.47	9.19	7.54	5.83	5.83	5.83	7.23	7.23	7.2	7.19	13.1	– 6.74	7.21	8.55	9.26
		(12.9)	(13.8)	(14.5)	(17.0)	(17.0)	(17.0)	(16.3)	(16.3)	(16.4)	(14.6)	(10.8)	(8.49)	(15.1)	(14.5)	(14)
55555	Shopping < weekly	– 1.14	– 1.05	– 1.32	– 1.22	– 1.22	– 1.22	– 1.22	– 1.22	– 1.23	– 1.46	– 0.897	– 1.48	– 1.6	– 1.41	– 1.76
		(2.2)	(2.18)	(2.17)	(2.15)	(2.15)	(2.15)	(2.16)	(2.16)	(2.15)	(2.13)	(2.1)	(2.08)	(2.14)	(2.14)	(2.15)
	Shopping 2–6 times/week	0.0627	0.121	– 0.0458	– 0.227	– 0.227	– 0.227	– 0.213	– 0.213	– 0.18	– 0.541	0.367	– 0.602	0.0676	– 0.265	– 0.203
		(2.21)	(2.19)	(2.18)	(2.16)	(2.16)	(2.16)	(2.16)	(2.16)	(2.16)	(2.14)	(2.1)	(2.1)	(2.15)	(2.16)	(2.16)
	Shopping weekly	– 0.207	– 0.034	– 0.108	0.0746	0.0746	0.0746	– 0.0408	– 0.0408	– 0.0481	– 0.497	0.0719	– 0.112	– 0.27	– 0.199	– 0.178
		(1.93)	(1.91)	(1.9)	(1.89)	(1.89)	(1.89)	(1.9)	(1.9)	(1.9)	(1.87)	(1.84)	(1.82)	(1.87)	(1.88)	(1.88)
	Shopping daily	5.73	5.23	5.41	5.03	5.03	5.03	4.94	4.94	4.93	5.15	7.11*	5.02	4.77	4.85	5.56
		(3.62)	(3.55)	(3.54)	(3.49)	(3.49)	(3.49)	(3.49)	(3.49)	(3.49)	(3.48)	(3.4)	(3.47)	(3.47)	(3.47)	(3.49)
	Shopping > daily	15.3	15.3	15.4	15.7	15.7	15.7	15.5	15.5	15.5	15.2	16.5*	2.25	15.3	15.6	15.8
		(8.91)	(8.89)	(8.89)	(8.89)	(8.89)	(8.89)	(8.89)	(8.89)	(8.89)	(8.75)	(8.35)	(9.93)	(8.76)	(8.75)	(8.75)
55555 rescaled	Shopping < weekly	– 1.35 × 10–3	– 1.57 × 10–3	0.0103	– 0.0111	– 0.0111	– 0.0111	– 0.195	– 0.195	– 0.178	– 0.0566	– 0.0217	– 0.0327	– 0.0316	8.19 × 10–3	0.0159
		(0.0279)	(0.0317)	(0.0361)	(0.0368)	(0.0368)	(0.0368)	(0.154)	(0.154)	(0.214)	(0.0844)	(0.0744)	(0.0763)	(0.0545)	(0.0427)	(0.0379)
	Shopping 2–6 times/week	2.44 × 10–3	– 0.0246	– 0.0179	– 0.0415	– 0.0415	– 0.0415	– 0.149	– 0.149	– 0.326	– 0.0317	– 0.0191	2.32 × 10–3	– 4 × 10–2	– 0.0191	2.5 × 10–3
		(0.0282)	(0.0318)	(0.0362)	(0.0369)	(0.0369)	(0.0369)	(0.155)	(0.155)	(0.214)	(0.0849)	(0.0746)	(0.0772)	(0.0547)	(0.043)	(0.0381)
	Shopping weekly	– 3.35 × 10–4	– 8.74 × 10–3	7.11 × 10–3	3.53 × 10–3	3.53 × 10–3	3.53 × 10–3	– 6.34 × 10–3	– 6.34 × 10–3	0.0329	– 0.0292	– 0.0527*	– 0.0515	– 4.07 × 10–3	2.75 × 10–3	0.0143
		(0.0245)	(0.0278)	(0.0316)	(0.0324)	(0.0324)	(0.0324)	(0.136)	(0.136)	(0.189)	(0.0742)	(0.0653)	(0.0668)	(0.0478)	(0.0375)	(0.0332)
	Shopping daily	0.0623	0.0177	0.0591	0.0116	0.0116	0.0116	– 0.0779	– 0.0779	– 4.17 × 10–3	0.0805	0.0662	0.0451	– 0.0193	– 0.025	0.0637
		(0.0466)	(0.0522)	(0.0596)	(0.0604)	(0.0604)	(0.0604)	(0.252)	(0.252)	(0.35)	(0.14)	(0.123)	(0.129)	(0.0895)	(0.0699)	(0.0624)
	Shopping > daily	0.156	0.161	0.191	0.201	0.201	0.201	0.265	0.265	0.294	0.142	0.093	– 0.0689	0.221	0.187	0.187
		(0.116)	(0.133)	(0.152)	(0.157)	(0.157)	(0.157)	(0.662)	(0.662)	(0.919)	(0.36)	(0.307)	(0.371)	(0.231)	(0.18)	(0.159)

*Note.* Standard errors in parentheses; * = significant at 5% level

**Table 14:** Rescaled 55555 robustness tests for Tobit regression results including frequency of leaving the house for exercise and fresh air

		Low rescaled 55555			Censoring rescaled 55555			Impact on mean rescaled 55555			High VAS for dead			Rescaled 55555 high rate of change		
		– 1	– 1.5	– 2	– 1	– 1.5	– 2	1.5	2	2.5	75	50	25	0.1	0.075	0.05
11111	Exercise < weekly	– 3.62	– 3.93	– 3.99*	– 4.29*	– 4.29*	– 4.29*	– 4.29*	– 4.29*	– 4.29*	– 4.33*	– 3.67	– 3.82	– 4.44*	– 3.91*	– 3
		(2.06)	(2.03)	(2.01)	(2.02)	(2.02)	(2.02)	(2.02)	(2.02)	(2.02)	(2.04)	(2.14)	(2.19)	(1.97)	(1.95)	(1.88)
	Exercise 2–6 times/week	– 1.58	– 1.61	– 1.76	– 1.68	– 1.68	– 1.68	– 1.73	– 1.73	– 1.72	– 1.84	– 1.98	– 1.72	– 2.43	– 2.43	– 2.13
		(1.72)	(1.7)	(1.68)	(1.69)	(1.69)	(1.69)	(1.7)	(1.7)	(1.69)	(1.71)	(1.76)	(1.8)	(1.64)	(1.62)	(1.55)
	Exercise weekly	– 4.58*	– 4.65*	– 4.63*	– 4.65*	– 4.65*	– 4.65*	– 4.8*	– 4.8*	– 4.8*	– 4.79*	– 5.33*	– 5.01	– 5.35*	– 4.56*	– 3.73*
		(2.03)	(2.00)	(1.98)	(1.98)	(1.98)	(1.98)	(1.99)	(1.99)	(1.99)	(2.02)	(2.1)	(2.18)	(1.93)	(1.92)	(1.84)
	Exercise daily	– 1.85	– 1.96	– 2.02	– 2.15	– 2.15	– 2.15	– 2.15	– 2.15	– 2.15	– 2.28	– 2.49	– 2.79	– 2.39	– 2.27	– 1.76
		(1.72)	(1.7)	(1.69)	(1.69)	(1.69)	(1.69)	(1.69)	(1.69)	(1.69)	(1.71)	(1.76)	(1.79)	(1.64)	(1.62)	(1.55)
	Exercise > daily	3.6	3.25	3.18	2.59	2.59	2.59	2.64	2.64	2.63	2.54	3.13	3.67	2.5	2.41	2.38
		(2.64)	(2.6)	(2.6)	(2.59)	(2.59)	(2.59)	(2.59)	(2.59)	(2.59)	(2.61)	(2.67)	(2.81)	(2.52)	(2.48)	(2.37)
Dead	Exercise < weekly	10.4*	11*	11.1*	12.2*	12.2*	12.2*	12*	12*	12.1*	11.9*	7.62*	3.33	11.5*	11.1*	8.66*
		(3.98)	(4.22)	(4.34)	(5.04)	(5.04)	(5.04)	(4.8)	(4.8)	(4.84)	(4.34)	(3.57)	(1.8)	(4.61)	(4.44)	(4.37)
	Exercise 2–6 times/week	0.83	1.47	0.983	1.68	1.68	1.68	0.528	0.528	0.943	1.19	3.2	-0.0619	2.78	2.46	1.3
		(3.44)	(3.65)	(3.75)	(4.37)	(4.37)	(4.37)	(4.17)	(4.17)	(4.21)	(3.77)	(3.02)	(1.53)	(3.97)	(3.82)	(3.71)
	Exercise weekly	9.63*	11.5*	11.3*	14.1*	14.1*	14.1*	12.8*	12.8*	12.8*	11.4*	8.33*	1.92	14.1*	12*	10.4*
		(3.95)	(4.16)	(4.28)	(4.96)	(4.96)	(4.96)	(4.73)	(4.73)	(4.78)	(4.31)	(3.52)	(1.82)	(4.51)	(4.38)	(4.28)
	Exercise daily	1.16	2.8	2.02	5.28	5.28	5.28	5.07	5.07	5.02	3.03	2.91	0.556	6.03	4.48	3.44
		(3.46)	(3.66)	(3.77)	(4.36)	(4.36)	(4.36)	(4.16)	(4.16)	(4.19)	(3.78)	(3.03)	(1.53)	(3.97)	(3.83)	(3.72)
	Exercise > daily	5.23	6.88	6.31	6.67	6.67	6.67	6.98	6.98	7.08	7.53	8.8*	1.16	7.85	7.66	6.75
		(5.1)	(5.38)	(5.6)	(6.5)	(6.5)	(6.5)	(6.19)	(6.19)	(6.25)	(5.56)	(4.32)	(2.31)	(5.89)	(5.66)	(5.5)
55555	Exercise < weekly	4.07	4.29	3.99	3.89	3.89	3.89	3.87	3.87	3.87	3.43	1.84	0.437	3.38	3.46	2.69
		(2.67)	(2.65)	(2.63)	(2.61)	(2.61)	(2.61)	(2.61)	(2.61)	(2.61)	(2.58)	(2.58)	(2.55)	(2.61)	(2.61)	(2.64)
	Exercise 2–6 times/week	2.59	2.5	2.71	2.59	2.59	2.59	2.62	2.62	2.65	2.62	3.1	1.78	2.86	2.95	2.92
		(2.22)	(2.21)	(2.19)	(2.18)	(2.18)	(2.18)	(2.18)	(2.18)	(2.18)	(2.16)	(2.11)	(2.08)	(2.17)	(2.17)	(2.17)
	Exercise weekly	7.53*	7.3*	7.42*	7.22*	7.22*	7.22*	7.47*	7.47*	7.47*	7.06*	6.32*	5.26*	7.86*	7.24*	7.38*
		(2.63)	(2.6)	(2.59)	(2.56)	(2.56)	(2.56)	(2.56)	(2.56)	(2.56)	(2.55)	(2.53)	(2.53)	(2.55)	(2.57)	(2.58)
	Exercise daily	2.48	2.51	2.76	2.78	2.78	2.78	2.83	2.83	2.82	2.76	2.73	1.13	3.15	3.03	3.12
		(2.23)	(2.21)	(2.2)	(2.19)	(2.19)	(2.19)	(2.18)	(2.18)	(2.18)	(2.16)	(2.12)	(2.08)	(2.18)	(2.18)	(2.18)
	Exercise > daily	0.0398	0.175	0.349	0.531	0.531	0.531	0.426	0.426	0.43	0.619	0.876	– 2.44	0.645	0.707	0.679
		(3.37)	(3.34)	(3.34)	(3.31)	(3.31)	(3.31)	(3.31)	(3.31)	(3.31)	(3.27)	(3.16)	(3.2)	(3.29)	(3.29)	(3.29)
55555 rescaled	Exercise < weekly	0.0125	9.41 × 10–4	– 0.0102	– 0.0155	– 0.0155	– 0.0155	– 0.0514	– 0.0514	– 0.0442	– 0.138	6.08 × 10–3	1.92 × 10–3	– 0.0127	– 0.0169	2.95 × 10–3
		(0.034)	(0.0387)	(0.0438)	(0.0449)	(0.0449)	(0.0449)	(0.187)	(0.187)	(0.26)	(0.102)	(0.0912)	(0.0937)	(0.0667)	(0.0522)	(0.0466)
	Exercise 2–6 times/week	0.0325	0.0245	0.0294	0.0255	0.0255	0.0255	0.0875	0.0875	– 0.0396	0.0237	0.014	0.0202	4.48 × 10–3	0.0185	0.0407
		(0.0282)	(0.0321)	(0.0364)	(0.0374)	(0.0374)	(0.0374)	(0.156)	(0.156)	(0.217)	(0.0854)	(0.0746)	(0.0762)	(0.0553)	(0.0432)	(0.0383)
	Exercise weekly	0.031	– 3.74 × 10–3	– 1.68 × 10–3	– 0.0251	– 0.0251	– 0.0251	– 3.78 × 10–3	– 3.78 × 10–3	0.0139	– 0.0244	0.0177	0.072	– 0.0299	– 0.0114	0.0237
		(0.0337)	(0.0381)	(0.0433)	(0.0442)	(0.0442)	(0.0442)	(0.184)	(0.184)	(0.257)	(0.101)	(0.0902)	(0.0936)	(0.0655)	(0.0515)	(0.0458)
	Exercise daily	0.0356	0.0123	0.0254	– 5.47 × 10–3	– 5.47 × 10–3	– 5.47 × 10–3	– 0.189	– 0.189	– 0.159	– 0.0393	– 0.0265*	– 0.0345	– 0.0523	– 6.46 × 10–3	0.0149
		(0.0283)	(0.0321)	(0.0365)	(0.0373)	(0.0373)	(0.0373)	(0.155)	(0.155)	(0.217)	(0.0855)	(0.0748)	(0.0762)	(0.0553)	(0.0433)	(0.0384)
	Exercise > daily	6.33 × 10–3	– 0.0125	– 3.24 × 10–3	– 0.0131	– 0.0131	– 0.0131	– 6.31 × 10–4	– 6.31 × 10–4	– 0.0222	– 0.0552	– 0.0323	– 0.023	– 0.0119	– 0.0116	5.35 × 10–3
		(0.0425)	(0.0482)	(0.055)	(0.0563)	(0.0563)	(0.0563)	(0.235)	(0.235)	(0.327)	(0.128)	(0.111)	(0.116)	(0.0833)	(0.0651)	(0.0576)

*Note.* Standard errors in parentheses, *significant at 5% level

## Footnotes

1 www.coronavirus.data.gov.uk.

2 https://api.coronavirus.data.gov.uk/v2/data?areaType=region&metric=cumCasesByPublishDateRate&metric=cumDeaths60DaysByDeathDateRate&format=csv for regions of England. https://api.coronavirus.data.gov.uk/v2/data?areaType=nation&metric=cumCasesByPublishDateRate&metric=cumDeaths60DaysByDeathDateRate&format=csv for Scotland, Wales and Northern Ireland.

3 https://euroqol.org/eq-5d-instruments/eq-5d-5l-about/valuation-standard-value-sets/new-uk-eq-5d-5l-valuation-study_blog/ accessed 8/2/22.
